# The global landscape of cognition: hierarchical aggregation as an organizational principle of human cortical networks and functions

**DOI:** 10.1038/srep18112

**Published:** 2015-12-16

**Authors:** P. Taylor, J. N. Hobbs, J. Burroni, H. T. Siegelmann

**Affiliations:** 1 College of Information and Computer Sciences. University of Massachusetts, Amherst, MA, USA; 2 Neuroscience and Behavior Program. University of Massachusetts, Amherst, MA, USA

## Abstract

Though widely hypothesized, limited evidence exists that human brain functions organize in global gradients of abstraction starting from sensory cortical inputs. Hierarchical representation is accepted in computational networks, and tentatively in visual neuroscience, yet no direct holistic demonstrations exist *in vivo*. Our methods developed network models enriched with tiered directionality, by including input locations, a critical feature for localizing representation in networks generally. Grouped primary sensory cortices defined network inputs, displaying global connectivity to fused inputs. Depth-oriented networks guided analyses of fMRI databases (~17,000 experiments;~1/4 of fMRI literature). Formally, we tested whether network depth predicted localization of abstract versus concrete behaviors over the whole set of studied brain regions. For our results, new cortical graph metrics, termed *network-depth*, ranked all databased cognitive function activations by network-depth. Thus, we objectively sorted stratified landscapes of cognition, starting from grouped sensory inputs in parallel, progressing deeper into cortex. This exposed escalating amalgamation of function or abstraction with increasing network-depth, globally. Nearly 500 new participants confirmed our results. In conclusion, data-driven analyses defined a hierarchically ordered connectome, revealing a related continuum of cognitive function. Progressive functional abstraction over network depth may be a fundamental feature of brains, and is observed in artificial networks.

Neuronal processes which create symbolic meaning from sensory transduction, awareness from neuronal spiking, and represent information in network structure, all remain deep neuroscientific mysteries, and lucrative challenges in artificial intelligence. The location of sensory inputs into large networks may impact functional representation (Fig. 1a). Computational connectionist structures like neural networks emergently yield hierarchical abstractions progressing from inputs (Fig. 2a). In many-layer artificial networks, popularized by deep-learning algorithms^1^, simple features manifest in layers nearer to inputs or sensors, while abstract representations emerge in deeper layers. Spatially progressive increases in amalgamation of representation or function emerge as distance from input increases^2^. This appears to evolve in numerous information processing connectionist architectures with more frequent connections between adjacent layers than non-adjacent layers, and where learning is distributed; mammalian cortices share these features. Networks with such properties, and adaptivity, feedback, or recurrence (e.g., brains) may blunt or enhance, not likely eliminate, this aggregation of function. It is important to question whether biological spiking networks generally sharing these simple sufficient features with artificial networks enables emergence of grossly similar information representation distributions in brains. Though adaptive information processing neural networks do not model brain complexity entirely, they appear to share some features based on their network structure, as we discuss below.

Studies of brain functions and behaviors exploring relationships between network representation, function, and structure compose the heart of neuroscience. Many previous studies theorized that information representation in the brain demonstrates a hierarchical structure, with simple sensations and perceptions processed near inputs to cortex, and more abstract, symbolic, or deep concepts processed more distant from sensory inputs, or deeper in the brain’s structural network. This has been occasionally simplified pictorially as a pyramid representing the global structure of cognition, with sensory processing at it’s foundational base, and abstract concepts and reasoning at the pinnacle. Pioneering visual stream research inspired models of hierarchical regionalization of brain functions^3^,^4^, with regions closer to initial sensory inputs activated by simple tangible features (e.g., lines, colors) and regions further from sensory inputs manifesting feature conjunctions and object representations. These principles are supported by biologically realistic models of object perception^4^,^5^,^6^,^7^,^8^,^9^ as well as by models of visual attention, directly^10^,^11^,^12^,^13^,^14^ and indirectly^15^,^16^. Similar but not identical functional principles are hypothesized for the auditory modality^17^,^18^, other senses such as the olfactory modality^19^, and commonly predicted for cortex as a whole, though without direct global experimental evidence.

To answer such large-scale questions, cross-discipline synthetic computational methods were required. Neuroinformatics and databasing approaches have greatly improved understanding of both the structural connectivity of the brain^20^, and of the functional specialization of brain regions^21^,^22^,^23^,^24^. This is often measured via functional magnetic resonance imaging (fMRI), elaborated in full depth below. Enabled by databases like these, we were thus inspired to search for globally intertwined hierarchies, both in cortical structure, and in cognitive function. Rather than attempt to bias or narrowly predict results, these studies were designed with a descriptive approach, using big behavioral fMRI databases and large-scale connectivity data, validating hypotheses afterwards via large-scale human surveys. We employed a novel method of illustrating geometrical distributions of behavioral fMRI activations in relation to any regional node-wise graph statistics of connectome data. Such global mapping enabled holistic insights to detect the tectonic patterns of thought and organization in the brain.

We hypothesized that regions deeper in the brain represent more abstract functions. We formally tested the prediction that region-wise degree of connectivity to inputs correlated with the abstractness of studied brain functions performed by those regions, with concrete functions appearing in areas most connected to inputs, and abstract functions appearing in those least connected to inputs, or deepest in cortex.

The structure of our paper is organized as follows: Methods describe the physiological connectivity matrices, the behavioral databases, and the algorithm of sorting these behavioral fMRI data by network-depth. Results illustrate brain regions via our novel graph metric, behavioral fMRI rankings by network-depth, two types of control experiments, and human survey data showing similar sorting via “abstractness” compared to our physiological methods, after which we provide further discussion and conclusions.

## Methods

### Executive summary of methods

Since the brain is so highly recurrent, it had been assumed that specifying a global, directional hierarchy would not be feasible, since interconnectedness would, in effect, disguise it. Relating the spectrum of cognitive functions to global cortical architecture required developing new approaches to modeling structural hierarchies. Hierarchy is traditionally defined as: *an arrangement, categorization, system, or series of objects (items, names, values, categories, people, groups, subsets, etc.) in which the nodes are ranked, organized, or arranged as being “above,” “below,” or “connected to” one another either directly or indirectly, either vertically or diagonally, in a graded or successive order or class, often according to importance, relevance, category inclusiveness, or according to similarities of structure or origin, all of which can be represented formally, usually by a diagram of connected nodes.*

Our formal definition of hierarchy (or graph-theoretic network depth) simplified recurrence into a continuum of statistical connectivity to sensory inputs. This notion of hierarchy was merely probabalistic, where depth was assumed to impact the probability of neuronal routing, rather than necessitate it. Our methods defined a structural pyramid of the human connectome, with the bottom foundation of the pyramid defined as sensory inputs, while the peak or pinnacle of the pyramid was structurally deepest in the cortical network. We associated every region of interest (ROI) in the brain with a number identifying its integrated distance from the average of sensory cortex inputs based on rsfMRI and DTI data, defined below. This defined connectivity-distance of that region from the sensory outside world, or that region’s depth in the cortical network, termed *network-depth*.

Next, we generated a novel method of describing the distribution of behavioral fMRI activations via their relationship with any node-wise graph statistic of connectome data. Finally, with this method we rank-sorted cognitive functions from fMRI databases by their distribution over network-depth, with cognitive functions distributed shallow in the structural network at the top of the list, and functions distributed deeply at the bottom of the list. As a proof of concept, we compared the physiologically-based ranking to a survey of humans’ ranking; around 500 participants were asked to rank behavioral elements by their judgment of whether they were “abstract” versus “concrete”. Further, we compared experimental orders to similar random control experiments, and to meaningful control experiments which sorted functions by other node-wise graph statistics. Some of our methods themselves were novel contributions, and were thus elaborated in full detail below.

### Connectivity methods

Two modern methods are popularly used to map the human brain’s wiring diagram, the connectome, in a living person: **1)** resting state functional magnetic resonance imaging (rsfMRI) measuring temporal correlation (functional connectivity) between regions; and **2)** diffusion tensor imaging (DTI) measuring tract-based connectivity between regions through fiber bundles from which distances can be calculated. With rsfMRI, a seed region (starting reference point) is selected, revealing to what degree other regions respond in temporal synchronization with that reference region, thus allowing inference about the degree to which brain regions are connected. The rsfMRI data serve as a proxy for processing delay between regions or number of functional synapses between regions, though are limited to peri-second resolution. DTI does not share the aforementioned temporal limitation, yet is neither functionally based, nor evenly distributed in signal resolution across the brain. Network models derived from DTI and rsfMRI demonstrate similarities and strongly correlate^25^. Big data connectomics contributed modern approaches, including repositories of connectivity for human cortex such as the 1000 Functional Connectomes Project, and the Human Connectome Project^26^. We extended and complemented the use of such connectivity data.

### Building connectivity matrices from rsfMRI and DTI data

Our network analyses utilized rsfMRI and DTI connectivity matrices from the same group of individual participants from the NKI Rockland sample^20^ hosted in the 1000 Functional Connectomes Project, the International Neuroimaging Data-Sharing initiative, and the UCLA Multimodal Connectivity Database (UMCD)^25^. Data processing for rsfMRI and DTI data followed procedures previously published^20^. Cortex, sub-cortical structures, and brain-stem were detailed. The rsfMRI data included negative correlations, which still indicate functional connectedness, and thus |*absolute*| value of that full matrix was used. Each full connectivity matrix was normalized between 0 (least connected) to 1 (most connected); by definition, normalizations do not change later rank-ordering. Diagonals of matrices were maximum values of the matrix, since regions are connected to themselves. The rsfMRI matrices were divided into 188 clusters (Regions-Of-Interest; ROIs), using a published parcellation process^27^ with resulting clusters imposed upon DTI data to parcel both data sets identically.

An increase in connectivity derived from rsfMRI + DTI is hereinafter termed *connectivity-distance*. Importantly, the *connectivity-distance* from any seed region may serve as a proxy for an increase in number of sequential synapses, processing time, or general connectedness from the seed region to any other region.

### Defining sensory inputs

**Figure 1:**
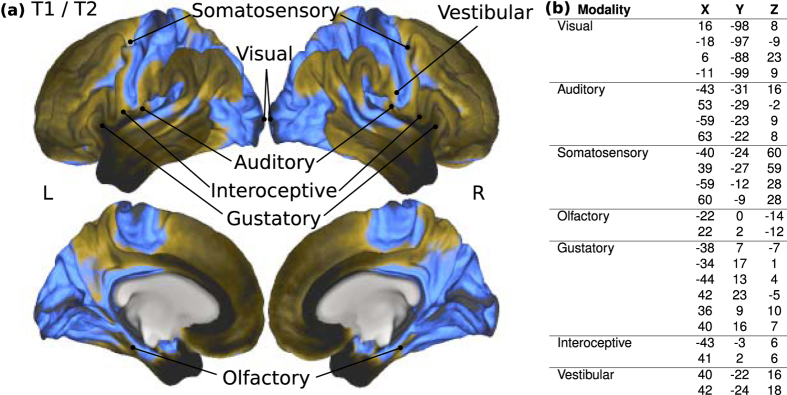
Inputs to the human cortical network. (**a**) Primary sensory cortices on T1/T2 MRI, indexing myelin density, generally greatest in sensory areas. Most senses relay through thalamus before cortex, except olfactory, connecting directly to cortex. All senses project bilaterally, except vestibular, being right lateralized for right handed individuals. (**b**) Input (seed) coordinates. For each given modality, if any of the post-parcellation clusters contained these tablular coordinates, that cluster was included as part of the aggregate seed for that modality.

First, we specified inputs to cortex. Information from the outside world is transduced via the senses, and information first reaches cortex in regions called “primary sensory cortices”, usually devoted to initial processing and perception of data within a single modality. These were hypothesized to be the sensors (inputs) to the information processing network of the cortex. The structure of sensory cortices differs from association cortices, for example in local/distant connectivity ratios^28^ and gross myelin distributions (Fig. 1a).

To identify known input regions, we performed extensive meta-analyses and employed the Harvard-Oxford cortical-subcortical probabilistic atlas included in FMRIB Software Library (FSL), and Brodmann’s labels from the BrainMap Talairach client^29^,^30^. Coordinates were determined for primary sensory input locations for visual, auditory, somatosensory, gustatory (taste), olfactory (smell), interoceptive (gut feelings), and vestibular (balance) modalities (Fig. 1). The vestibular modality can be subdivided into two modalities for detecting static head rotation (gravity) and dynamic momentum, and the interoceptive modality is not always considered a single modality nor a modality at all; however, both were included as single modalities in these analyses. The primary sensory cortices were labeled upon the T1/T2 map representing myelin distribution (Fig. 1a), and coordinates (*x*, *y*, *z*) corresponding to the centers of several clusters for each modality were listed in (Fig. 1b). All processing employed Montreal Neurological Institute (MNI) space.

#### Vestibular

The sense of balance relays through the vestibular nuclei of the brain-stem, through thalamus, branching to an area of posterior parietal cortex overlapping with Brodmann area 5 and nestled near somatosensory cortex, superior temporal gyrus, and central suclus^31^,^32^. Vestibular cortex displays the greatest degree of lateralization between hemispheres when compared to other modalities^33^,^34^.

#### Gustatory

Taste input relays through the gustatory nucleus of the solitary tract complex in the medulla, to the ventral posterior complex and the ventral posterior medial nucleus of the thalamus, on the way to primary gustatory cortex. Primary gustatory cortex overlaps with anterior insula and the frontal operculum on the inferior frontal gyrus of the frontal lobe^35^,^36^,^37^, and in part Brodmann area 43. These regions are often referred to as AIFO (Anterior Insula, Frontal Operculum).

#### Olfactory

The sense of smell is unique, as it is the only modality that does not primarily relay through the thalamus on the way to the cortex; projections are received directly to cortex. Targets include pyriform cortex, olfactory tubercle, amygdala, periamygdaloid cortex, and entorhinal cortex, roughly corresponding to Brodmann area 28 and 34^38^,^39^.

#### Interoceptive

Anterior insula is hypothesized to receive input from internal organs, and to play a roll in bodily awareness, emotional processing, physiological homeostasis, or more generally, interoceptive awareness^40^,^41^. This composes parts of Brodmann area 13.

#### Visual

The well-studied visual inputs relay through the lateral geniculate nucleus (LGN) of the thalamus to primary visual cortex (striate cortex or V1) and is traditionally defined as Brodmann area 17.

#### Auditory

Inputs for the sense of hearing transmit through brainstem (cochlear nucleus, superior olivary complex), inferior colliculi, then medial geniculate nucleus of the thalamus, before arriving at primary auditory cortex (A1). A1 roughly corresponds to superior temporal gyrus (STG), Heschl’s gyrus, as well as parts of planum polare and planum temporale. A1 overlaps with Brodmann area 41, some of Brodmann area 42, and is sometimes proposed to be in small part overlapping with Brodmann area 22.

#### Somatosensory

The well-studied inputs to cortex for the sense of touch relay through the brainstem to the ventral posterior nucleus of the thalamus before reaching the primary somatosensory cortex (postcentral gyrus). The primary somatosensory cortex overlaps primarily with Brodmann area 3a and 3b, though 2 and 1 may overlap partly as well.

### Connectivity Data processing

#### Degree of connectivity to each input

**Figure 2:**
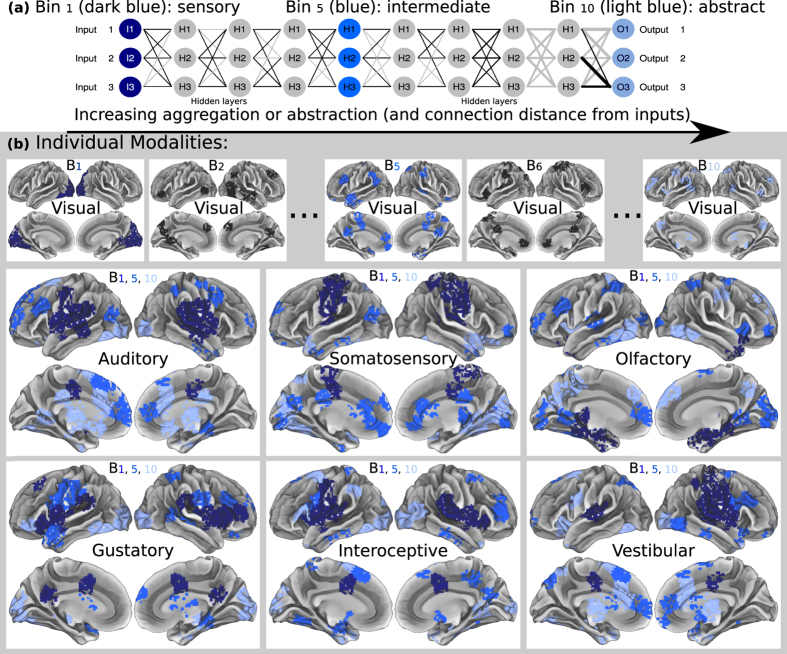
Network-depth via sensory inputs, as a proxy for gross directionality. (**a**) Conceptual plot showing emergence of hierarchical representation in a many-layer artificial network with inputs at left. Following left side input neurons (I) simple features typically emerge (dark blue) in hidden neurons (H); more aggregate features emerge more distant from inputs at right (blue, light blue), before right side output neurons (O). Colors match: (**b**) Regions discretized into 10 bins by network-depth. Top row illustrates bins progressing from visual cortex, left to right. Bottom two rows illustrate binned connectivity to remaining sensory cortices. Highlighted bins: *B*_1_ (dark blue) is most connected and closest to primary sensory cortices, *B*_5_ (blue) is somewhat connected/close to cortical inputs, *B*_10_ (light blue) is least connected and/or farthest away from cortical inputs.

**Figure 3:**
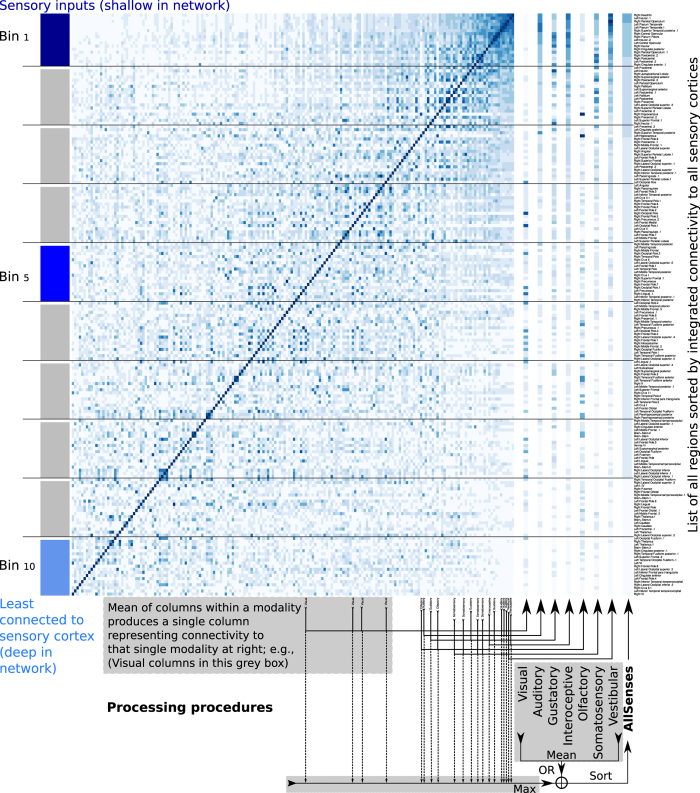
Defining generalized network-depth within cortex. Fully connected weighted symmetric matrices (DTI or rsfMRI) display sets of connectedness values, (188 x same 188), 1 for each major ROI. Connectivity for a single region was defined color in by 1, 1*188 row (or identically symmetric column). Mean for a single modality was generated by averaging multiple columns corresponding to regions within a single sensory cortex, displayed by arrows below the matrix; e.g., column labels and arrows for visual in left bottom grey box. Averaging resulted in 1 vector of length 188, representing the connectivity to a single modality, displayed as a wider column at the right of the matrix. To generate the cross-modality connectivity (global network-depth), 7 modalities (each represented by wide column at right) were integrated together (mean or max; bottom right grey boxes) to create 1 vector (far right widest column) defining connectivity of each brain region to all sensory inputs. This was used to sort the matrix along both dimensions. Cross-modality mean can be conceptualized as the average neuronal path length from any region to sensory cortices. Alternatively, cross modality maximum across all sensory columns was taken, representing the shortest neuronal path from each region to sensory seeds. Exemplar data illustrate rsfMRI with mean processing and sorting (plotted on the brain in Fig. 4 panel 1).

For each sensory area independently, we defined a starting seed (reference) region, and calculated its degree of connectivity to all other regions in cortex (Fig. 2b). Procedures follow: If primary sensory cortex input coordinates (Fig. 1) were fully within a cluster (out of 188), then the cluster was considered as a primary seed region (Fig. 3). Then, we averaged connectivity profiles from multiple seeds clusters of each single modality. (Fig. 3). This process revealed a continuum of regions, which were most to least connected to a sensory input, for each modality. Specifically, it generated a single vector representation (one value for every brain ROI) indexing the degree to which every other region is connected to that sensory seed input group. For example, connectivity to primary visual cortex was defined by averaging the connectivity to multiple seed clusters with centers in primary visual cortex (Fig. 1). This provided gross network-depth directionality for each single modality, thus defining our “hierarchical” rank.

Importantly, all remaining figures are color-coded identically, in gradients of blue representing network-depth, with shallow defined as dark blue, and deep defined as light blue.

#### Degree of connectivity to fused inputs

**Figure 4:**
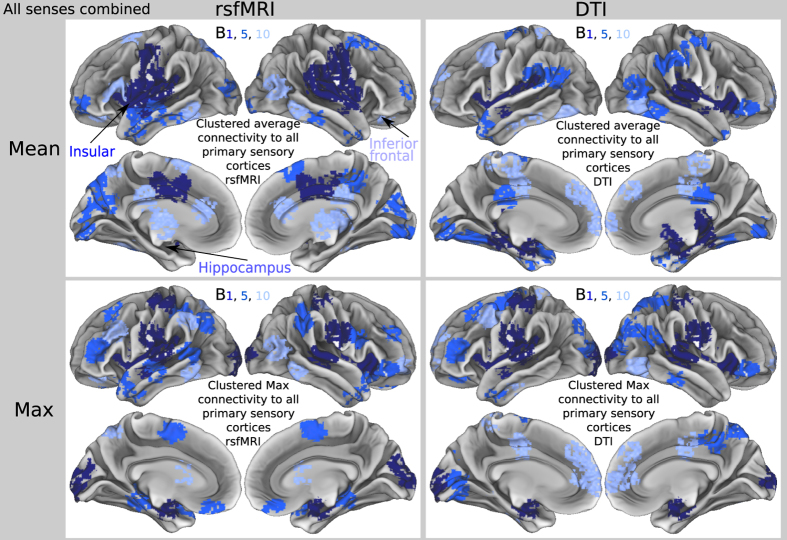
Network-depth via integrated sensory inputs displayed over whole-cortex. Brains display binned connectivity to an aggregate seed cluster of all grouped inputs in parallel, with DTI (brains on left) or rsfMRI (brains on right), using network mean (brains on top) or maximum (brains on bottom). In top left quadrant, key regions discussed in main text were labeled by respective color (e.g., insular cortex in dark blue text, and inferior frontal in light blue).

All modalities were examined in unison, since any behavior involves not only a single sensory input, but a combination of different senses. Above, we generated connectivity profiles for each modality independently first. Then, we integrated connectivity to all modalities (via max or mean), to provide a cross-modality connectivity dataset with equal weighting for each modality. The cross-modality seed was computed via either: 1) taking the mean across modalities, or 2) taking the maximum across modalities (Figs 3 and 4). Any ties during max comparison were broken by means. This aggregate seed composed of sensory regions illustrated connectivity of every region in cortex to sensory inputs, a proxy for global network-depth.

#### Binning network-depth: discretizing generalized connectivity-distance from sensory inputs

For the purpose of statistical summary, we divided the sorted distribution of connectivity-distance. Specifically, we artificially discretized sorting of regions via connectivity by distributing clusters into 10 step-wise statistical bins based on the degree of connectivity-distance of each ROI to input cortices. We divided the brain into 10 equally sized, irregularly shaped, groups of regions, ranging from highly connected to least connected, defining 10 steps of regional depth within the cortical network. The first clustered bin of ROIs, *bin*_1_ (dark blue in Figs 2, 3, 4), included sensory cortices and highly connected close regions, *bin*_2_ included proximate regions slightly less connected… *bin*_5_ (blue) included intermediately connected regions… and *bin*_10_ (light blue) included regions least connected to sensory inputs and deep in the brain’s network. It is important to note that these bins were not hypothesized to be layers or functional divisions, but were merely a method of descriptive analysis.

This process was performed for each modality and integrations of modalities (mean/maximum). The process of parceling into 188 clusters was performed before binning, thus facilitating correct super-clustering into bins, since bin divisions were more likely to occur between natural boundaries than within. Binning was performed evenly in region space, rather than connectivity space; each bin has a similar size. Plotting of images upon cortical surfaces^42^ was performed in Connectome Workbench^26^. To display this binned connectivity, the entire coordinate set from the BrainMap database was allotted into 10 bins and plotted to generate color-coded brain images (Figs 2b and 4).

The brain’s network only receives sense information via these sensory cortices and nowhere else. Thus, this measure estimates global network-depth within the brain.

### Behavioral fMRI methods

One popular method for investigating the localization of brain functions is fMRI, which measures changes related to blood flow, and thus indirectly, neuronal activity. Various experimental designs allow for inferences regarding activity of brain regions during behavioral tasks. Since fMRI studies are notoriously statistically under-powered or biased^43^,^44^, databasing many studies has strengthened potential reliability of conclusions^21^,^22^,^23^,^24^,^45^,^46^. Building upon a successful history of discovery in cognitive neuroscience using fMRI, our global synthesis included multiple large databases of studies, enabling exploration of larger-scale organizational principles of brains and behavior.

#### Determining functional localization from large behavioral fMRI databases

**Figure 5:**
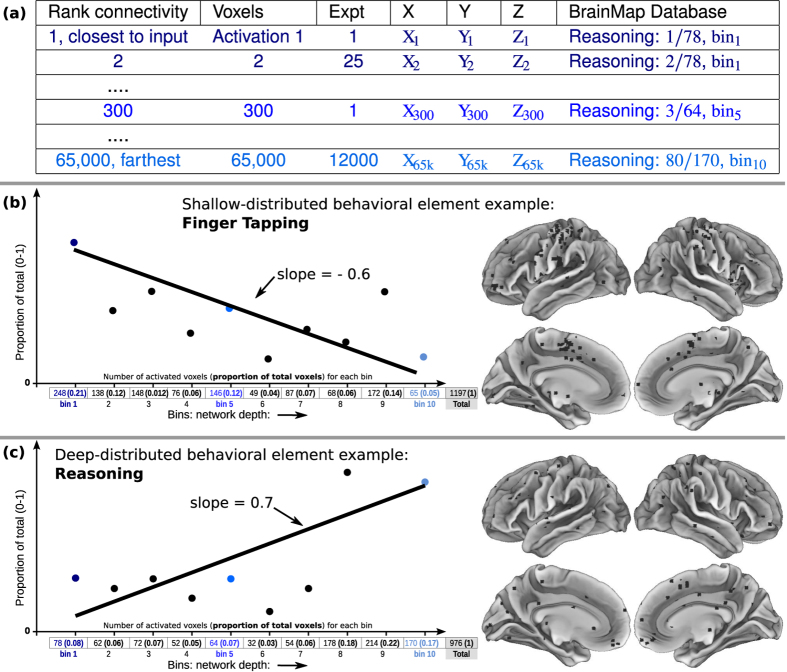
Binning behavioral-fMRI activation coordinates by physiological connectivity data. (**a**) Top table displays format of fMRI data, with behavioral elements tallied (at right of table) by sorted connectivity to inputs (at left of table). (**b**,**c**) Lower line-plots display examples of calculations performed for BrainMap and Neurosynth behavioral elements, with activation proportions illustrated from 0–1 on Y-axis, and network-depth sorted bins 1–10 on X-axis. Small table immediately below X-axes of the lineplots displayed each database’s behavioral elements summarized using the number of voxels activated per bin and the relative activation proportions (in parenthesis). Exemplar 1,197 and 976 activations were each derived from 151 and 53 experiments respectively. Binning was performed on sorted connectivity from inputs, with matching color codes for network-depth (Figs 2, 3, 4). For each behavioral element, a linear regression was calculated, with slope. Slope of activation proportions over bins provided a concise model of each behavioral element’s activation distribution relative to sensory inputs. Brains at right illustrate corresponding activations in brain space. Sensory-distributed behavioral element activations appeared more grouped, while deep-distributed appeared more dispersed.

**Figure 6:**
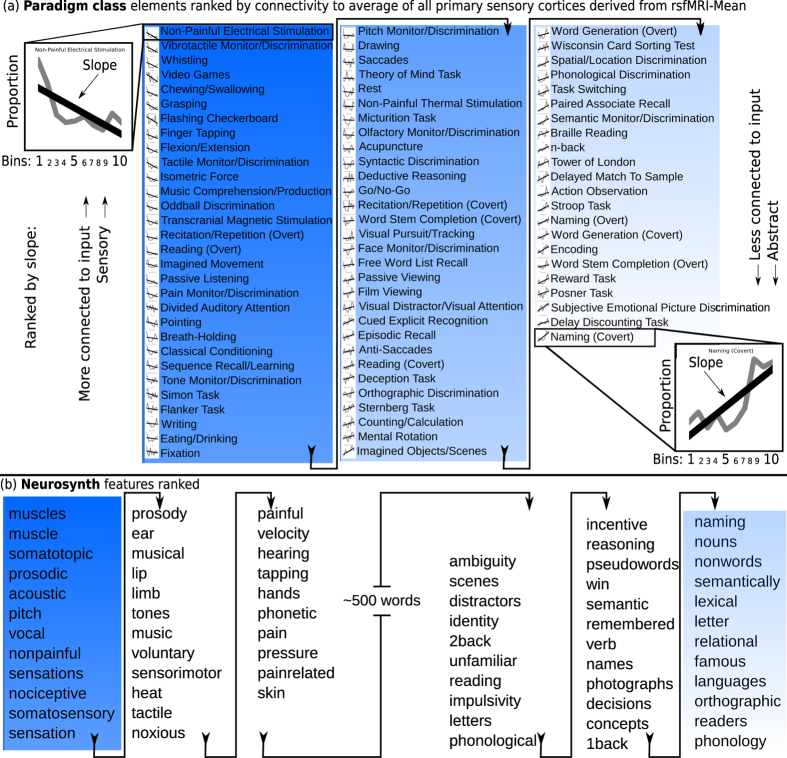
BrainMap paradigm class and Neurosynth behavioral elements ranked in order of slope via network-depth, progressing from all inputs concurrently across cortex. In auto-sorted lists, sensory-related behavioral elements emerged at the top, and aggregate symbolic tasks appeared near the list’s bottom, revealing an apparent relationship between behavioral element abstractness and network-depth. For example, compare the plots for the initial and final behavioral elements. (**a**) Every behavioral element from Paradigm Class auto-ranked in a sorted list. Interestingly, this linear regression merely approximated the actual distributions, which are all shown as small subsets next to each word. (**b**) Neurosynth behavioral elements auto-ranked; only terminal ends of list displayed (sensory and abstract ends). All other evaluated lists included in Supplementary Data Tables S2–4.

We combined our newly-directional cortical network models with large-scale behavioral data. For a fully-blind synthesis enabling objective results, we chose two different large repositories of behavior-associated fMRI activation coordinates. These repositories contain thousands of experiments and included:

1. BrainMap is a database of manually entered fMRI publications including activation coordinates associated with tasks, among other meta-data categories^21^,^22^,^23^,^24^. To date, it included 2390 papers, 11353 experiments, 46366 subjects, and 91039 coordinate locations. Studies are entered manually, and thus higher quality than automated extractions. The database is around 20 years old and hosted by Research Imaging Institute of the University of Texas Health Science Center San Antonio < http://www.brainmap.org>. We utilized two meta-data category labels: The *Behavioral Domain* label describes cognitive processes tested by fMRI contrasts in various experimental designs, composed of sub-elements such as: motion perception, anxiety, and memory. The *Paradigm Class* label describes the type of task performed, composed of sub-elements such as: grasping, whistling, and Stroop task. (Example data in Fig. 5a; Complete lists available in Fig. 6a; Supplementary Data Tables S2,S3).
2. The Neurosynth database auto-extracts tabular fMRI activation coordinates and word frequencies from published studies^45^,^46^. To date, it contained 200,000 activation coordinates derived from 6,000 publications, and is hosted at the University of Texas at Austin. Word frequencies (*features* in Neurosynth) auto-extracted from each article are associated with auto-extracted fMRI coordinates. This results in a set of features for each study, labeling a linked set of activation coordinates. For example, an article may use the word “faces” at a greater frequency than other articles, which is bound to coordinates reported by that article (a sample of Neurosynth word elements occur in Fig. 6b; complete list in Supplementary Data Table S3). Neurosynth data can be obtained via < http://neurosynth.org>, or via NeuroDebian^47^ repositories. Auto-extracted data are representative enough of actual human brain functions that this project enabled the beginnings of cursory computational ‘mind-reading’^46^, entailing the ability to predict basic states of mind using brain activations by comparing these states of mind to the database of known behavior-activation mappings.

Each single fMRI study in these databases associated the topic(s) of study of a single paper (e.g., face processing, visual cognition) with a set of regions which differed in activity between conditions, after statistical thresholding, for that single paper. Both database quality controls and analyses confirm external validity^21^,^46^. Together, these repositories tabulated activations from a potential ~17,000 fMRI experiments, with each individual study typically ranging from 500 mb to 5 gb per subject of raw data, summarizing potentially ~600 terabytes (0.6 petabyes). Notably, human input only indirectly shaped the results reported here via the studies that went into the fMRI databases and the publication process itself. There is some limited documented overlap in study indexing between databases. Since the Neurosynth database includes auto-extracted data, it likely contains some limited non-task-fMRI data, for example by incidentally indexing voxel-based morphometry (VBM) studies. In the BrainMap or Neurosynth databases, any task-based behavioral fMRI data in Talairach brain space were converted into MNI space using the icbm2tal transform^48^,^49^.

Importantly, each repository distinctly categorizes studies by one or several of about 650 employed labels (hereinafter: *behavioral elements*). Behavioral elements were conceptualized here as brain functions, tasks, or behaviors, which through various experimental designs elicited activations at corresponding coordinates. The data for each behavioral element included many points of activation from many studies.

#### Synthesizing physiological connectome with behavioral fMRI, via generalized network-depth from sensory inputs

We synthesized the above structural data and functional task-fMRI databases, to sort all behavioral elements by network depth. We objectively graphed distributions of activations associated with each behavioral element, across 10 bins of connectivity-distance to primary sensory cortices (Fig. 5). For each bin of network-depth, we summed all fMRI activation coordinate locations associated with each behavioral element showing activations in that bin, and calculated the proportion of activations out of the total. This was performed by dividing the number in each bin by the total in the brain, then normalizing, to generate a series of 10 proportions representing the relative brain activity corresponding to each behavior in each of the 10 bins (Fig. 5):


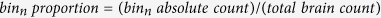


For example, one BrainMap behavioral element, ‘naming’, associated with 78 activation coordinates in *bin*_1_, 64 points in *bin*_5_… with 976 total brain activations; thus 78/976 = 0.08 for *bin*_1_ (dark blue)… 64/976 = 0.07 for *bin*_5_ (blue)… This process generated length-10 vectors describing distributions of activation proportion (Y-axis) over 10 bins (X-axis), for each behavioral element of meta-data categories (Fig. 5). Linear regressions approximating vectors were drawn; this line’s slope served as a concise model of the distribution of activations in sensory-connected bins (*bin*_1_…) versus activations in sensory-distant bins (…*bin*_10_). Thus, negative slopes described behavioral elements with activation distributions closer to sensory inputs, horizontal lines described distributions equally across bins, and positive slopes described distributions biased toward regions distant from inputs. In other words, slope is merely a numerical estimate of how connected to sensory inputs the entire set of activation coordinates for a single behavioral element were.

BrainMap (Paradigm Class and Behavioral Domain) data processing differed from Neurosynth, in that when counting activation coordinates in the Neurosynth database^46^, each feature loading or significance value (all less than 0.05) was used to slightly weight the count of each activation, such that features with higher probability were tallied more strongly. This was performed by tallying the inverse of each loading value, ranging from [0.95, 1]. If this range-modulated counting had any significant effect, it would be to slightly improve the accuracy of such a tallying method. Due to normalization and proportional analysis, our method was robust to biases in number of behavioral elements and publication bias present in the behavioral databases. Such bias has been elegantly illustrated by the article titled: “What is the most interesting part of the brain?”^43^. To adjust for any remaining sample size effects (varying experiment numbers per behavioral element) we tested our method with SlopeCoefficient * *γ* ln(Sample Size) with *γ* such that 0 < *γ* ln(Sample Size) ≤ 1, to push small samples toward a normalized mean of 0, which had minimal impact.

One benefit of bins is similar to that of spatial smoothing in fMRI analysis, eliminating high frequency noise, before we performed the linear regression. This process emphasizes global trends, which were the goal of our hypotheses. Before a linear regression, binning is optional. However, doing so is mathematically similar to not, and thus both would necessarily produce similar sorting above a reasonable number of bins (around roughly 6–7). Higher numbers of bins would mathematically approach a standard linear regression, while lower numbers would begin to lose important resolution. Above a threshold of bins, binning before regression would at most only blur any high-frequency patterns, rather than obscure or bias large-scale results. We ploted the identical methods with 47 bins in Supplementary Fig. S4.

### Algorithmic flow for data synthesis

General algorithmic procedure for plotting and sorting the behavioral databases upon connectivity matrices can be outlined in four steps: **(1)** Compute connectivity order, **(2)** Compute behavioral element frequencies, **(3)** Bin connectivity ordering, **(4)** Regress behavioral element frequency across bins, **(5)** Corrections on the set of slope coefficients.

1. ***Compute connectivity order***
  - Import a list of seed regions of interest (labeled sensory input locations, MNI space), and map these to the closest points in the connectivity matrix
  - Create an average (mean) of connectivities to input regions for each modality, resulting in one vector of dimension |*M*| * 1 per modality, named *V*_*M*_
  - Create an integration (mean or max) between each *V*_*M*_, creating vector *V*_*C*_, resulting in a single aggregate seed cluster for connectivity to all inputs
  - Sort *V*_*C*_ , and store its index for future ordering of behavioral elements
2. ***Compute behavioral element frequencies***
  - Import a behavioral database, *D* (Neurosynth or BrainMap) of points of activation and associated behavioral element labels, and map coordinates to the closest points in the matrix
  - For each behavioral element *E* ∈ *D*, generate a vector specifying the frequency of occurrence of coordinates associated with *E*, creating (*V*_*E*_)
  - For each coordinate entry in *D*, if the point corresponds to the current *E*, increment the corresponding index in *V*_*E*_ by the value 1 (BrainMap) or loading values ∈[0.95, 1] (Neurosynth)
3. ***Bin connectivity ordering***
  - Index and sort the frequency table of *V*_*E*_ using the previously computed connectivity order of *V*_*C*_
  - Split *V*_*E*_ into 10 bins of approximately equal size, *bin*_*n*_
  - Compute the sum of frequencies for each *bin*_*n*_
  - Weight bins of different size proportionally
4. ***Regress behavioral element frequency across bins***For each *E*:
  - Compute the proportion of activations for each *bin*_*n*_
  - Regress the proportion vs the bin index: 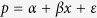 where *x* is the *bin*_*n*_ indices 1, 2, 3, …, 10, *p* is the proportion of activations, *ε* is the error term, *α* is the constant intercept term, and *β* is the slope. As per [50, p. 316] we applied the *logit transformation* to the proportions, 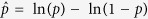 to obtain an appropriate distribution before regression.
  - Use the slope *β* to sort the behavioral elements
  - Plot the binned data and the regression
5. ***Corrections on the set of slope coefficients***
  - Conservative adjustment for behavioral element sample size variation via: SlopeCoefficient * *γ* ln(SampleSize) with *γ* such that 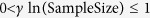, while ensuring that the data are normalized to 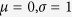 before correction.
  - z-score Normalization to facilitate cross-experiment and cross-method comparison and averaging

#### Random experiments

We defined two random experiments elaborated in more detail below. Each of the random experiments applies randomness to a different point in our algorithm.

1. Create a random index in [Compute connectivity order].d above
2. Sort randomly the bin index (*x*) in [Regress behavioral element frequency across bins].b above

### Human participants rank behavioral elements

To subjectively evaluate impressions of abstractness of behavioral elements, nearly 500 adults participated in our survey. As a recent trend in psychological and social sciences, research participants are often recruited from online services such as Amazon Mechanical Turk, or others including MicroWorkers, ClickWorker, CloudCrowd, and ClickChores. The population characteristics of these pools is similar to internet users generally, and the data quality is considered reasonably reliable due to screening and vetting processes employed by these services. The subject population for this study was recruited both locally from the university population and from a pool of online research survey participants via the company SocialSci (Cambridge, MA, USA). Participants all stated they were at least 18 years of age, fluent in English, and additionally reported details of any second language experience. All procedures complied with departmental and university guidelines for research with human participants and were approved by the University of Massachusetts Amherst Institutional Review Board.

All participants completed informed consent. Then, participants were initially provided with extensive definitions of: *concrete*, *abstract*, and *abstraction*, and asked to sort experimental phrases based on their judgment of whether they seem concrete versus abstract. Actual instructions were provided (Fig. S5). Two question formats included: 1) ranking a single phrase from 1-concrete to 7-abstract on a Likert scale, or 2) sorting 7 phrases in order of abstractness. To eliminate any order effects or biases, each participant received random samples of questions, presented in random order, both question types, and for sort-based questions received random intra-question order. Participants had no knowledge of the goals of the study, or our results.

Human participants’ behavioral element order of judged abstractness was analyzed via mean abstractness rank given to each word by participants (ranked 1–7 for both question types). Since surveys from unsupervised online participants are likely to contain some false or erroneous data due to attrition or technical failure, standard outlier exclusion (outer fences) was performed for each behavioral element, excluding around 2–3 subjects each, a conservative elimination. Relationships between network-depth based sort and normalized survey data were quantified via Pearson’s product moment correlation coefficient, and computed in the R core statistical environment. Aggregate ranks for each database-survey pairing were generated via mean behavioral element slope and order across all methods employed (DTI-Max, DTI-Mean, rsfMRI-Max, and rsfMRI-Mean) for each database independently.

For Paradigm Class and Behavioral Domain, all behavioral elements were provided to participants and included in analyses. For Neurosynth some behavioral elements appeared as apparent artifacts of the automated extraction process. Thus, ~50 out of 550 theoretically invalid Neurosynth features were excluded, including those which were: statistics-related, experimental-design-related, subject-population-related, neurological terminology, drugs, inappropriate to rank, or highly ambiguous; e.g., we excluded the terms: resting-state fMRI, significant, elderly, gene polymorphism, pharmacology, drug names, etc, since these are not actually topics of study, but artifacts of the word-frequency derived content in the Neurosynth database. These excluded words were chosen before running the survey, and we completed an identical analysis including these items; this conservative elimination did not appreciably impact statistical conclusions or sorts (Supplementary Data Tables S2–4).

## Results

### Executive summary of results

First, we built directionality into traditional human connectome models, furthering the canonical conceptualization of directional connectivity in neuroscience, as well as practice in computer science and graph theory. Then, we combined thousands of fMRI studies with connectivity models. This revealed an objective hierarchical landscape of cognition in the brain, with awareness at its structural core, and all results defined solely by a computational analysis, largely devoid of human bias. Specifically, our results illustrated two interrelated findings: 1) a structural network hierarchy based on cortical architecture, starting at sensory cortices, and extending to distant regions least connected to sensory input, and 2) a corresponding hierarchy of cognitive functions stretching from primary sensory relay and processing, to higher cognition. Finally, survey results indicated that our cognitive hierarchy based on network-depth aligned well with humans’ intuitions regarding information abstraction. Main results provided theoretical advances toward understanding the global distribution of information representation in cortical networks, as well as toward understanding functions of various brain regions based on connectivity to inputs.

### New cortical graph metric: network-depth defined as integrated connectivity-distance from all inputs

We hypothesized that a physical processing hierarchy would begin at or near sensory input, and proceed statistically. For the first time, we illustrated a whole-brain map of connectivity-distance from the combined group of seven sensory input cortices integrated into one reference seed (Fig. 4). Our cross-modal matrices represented connectivity of all ROIs, to the average of all inputs to cortex concurrently (Fig. 3). This improved illustrations of directionality in connectivity of human cortex by defining sensory cortices as network inputs.

To better describe the generalized network-depth measure proposed here, we explored the linear relationship between depth and other common node-wise graph measures. To analyze fully connected weighted matrices, graphs for DTI and rsfMRI were binarized, keeping 20% of the strongest connected edges (connections between nodes). This allowed other graph measures, requiring sparse connectivity, to be computed. Regions more shallow in the network tended to have greater *betweenness centrality* (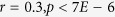; Pearson’s coefficient). A region with high betweenness or centrality must be passed through to get to other regions, and is often defined as the number of shortest paths from each vertex to all other nodes which pass through the seed. Regions with high betweenness centrality participate in many shortest paths. Regions more shallow in the network tended to have a greater *degree*, with more other regions connected to them, compared to regions deeper in the network, which had fewer connections on average (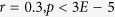). Degree was defined as the number of edges connecting to the node. Regions more shallow in the network tended to have greater *regional efficiency* (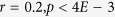). Regional efficiency described indirectly how connected a given vertex was to each other vertex in the network. Regions with higher efficiency measures had shorter minimum path lengths to each other node. We also analyzed network depth over the maximum spanning tree (Supplementary Fig. S1,2). The Maximum Spanning Tree (MST) was defined as the set of links and nodes spanning the graph which connect all vertices with maximum weight. Such visualizations are often used to derived conclusions from complex graphs^51^.

Our network-depth as a statistic applies only to graphs where inputs are known, which may be particularly relevant for neuronal networks, artificial and biological. Just as the geographic location of sources of raw materials would inform the placement of manufacturing facilities, our network-depth measure may be useful for explaining the functional specialization of regions of cortex.

### Ranking behavioral elements to depict the structure of cognition via tiered network-depth from inputs

We constrained behavioral activation coordinates from ~17,000 fMRI studies using our enriched hierarchical connectivity model. We sorted behavioral elements by associated activation proportion slopes (Fig. 5) with exceptional results (Figs 6). Use of slope assumed that a behavioral element’s activation was not limited to one location, but that even abstract behaviors start with sensory inputs, perhaps activating wide swaths of brain. We also assumed some small degree of linearity in functional distribution. Rather than obscure from the reader the actual distributions, many were shown as small insets left of each behavioral element (Fig. 6a).

Results were formatted as ranked lists of behavioral elements with activation distributions biased toward input regions at the top of lists, and behavioral elements biased toward deeper network regions lower in lists. Using our directed model representing average connectivity to all sensory inputs, results follow for the peripheral ends of the ranked lists. Sensory and Deep-network ends of the lists are detailed here:

#### Sensory distributed

Behavioral elements with slopes distributed toward sensory regions included those such as: “somatosensory stimulation”, “basic sensation”, “muscular”, “vibrotactile discrimination”, and “whistling”.

#### Abstract distributed

Behavioral elements with slopes biased towards the deep network extremity include abstract symbolic tasks such as: “reasoning”, “naming”, “concepts”, “language”, “cognition”, “imagination”, and “humor”.

Both distinct fMRI databases appeared to auto-sort as predicted by our *symbolic continuity hypothesis*. Our brain network models, with directionality nascently enabled by defining sensory inputs, revealed potential gradients of abstraction-related functional activation across cortex. Behavioral elements biased toward brain areas most connected to the outside world, at the base of the pyramid, were related to simple perceptions, sensory processing, or physical actions. On the other hand, behavioral elements biased toward brain areas least connected to the outside world, deepest in the brain’s network, and farthest from inputs, were related to more abstract concepts and symbols. For example “humor” appeared as the most abstract behavioral element in BrainMap Behavioral Domain (Supplementary Data Table 1). Another example, “n-back”, a working memory task which has been studied in relation to intelligence quotients (IQ), was distributed deeply in cortex. Overall, those regions at the pinnacle of the hypothetical pyramid model were objectively demonstrated to perform what appear to be hierarchically higher, more abstract, or deeper functions of mind.

This relationship between behavioral fMRI data and network geometry is novel and elegantly descriptive. Notably, different procedures used to generate each repository contribute to the external validity of any conclusions drawn from our studies, which interestingly replicated across databases and processing methods.

To generalize this method, it should be noted that if a linear regression does not seem ideal, it is possible to apply many different methods of fit to sort each behavioral element (e.g., a fit based on a quadratic function). In addition this sort method can be employed on virtually any regional graph statistic (e.g., regional efficiency instead of network-depth), as we did for an additional control (described below).

### Quantifying the pseudo-subjective evaluation of behavioral element rank order

**Figure 7:**
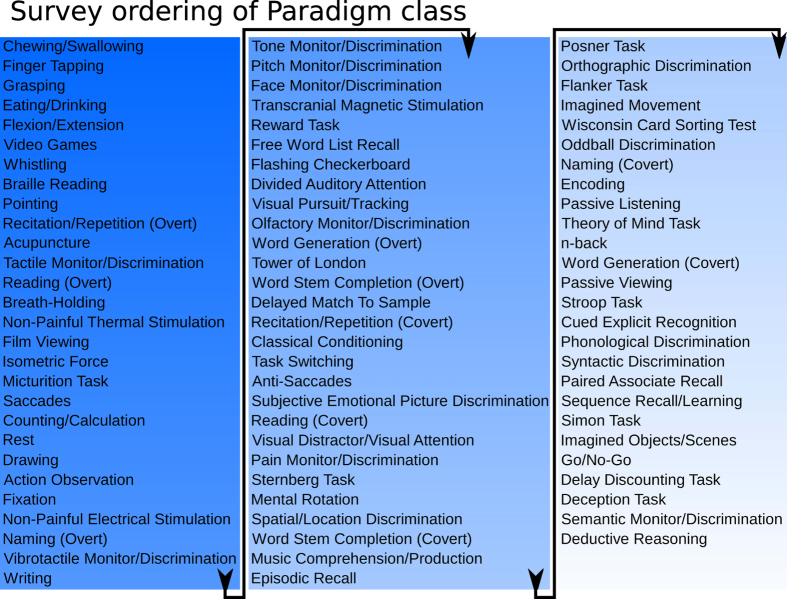
Example of the paradigm class behavioral element rank ordering via evaluation by ~500 survey participants. Words near the top of the list appeared to be ranked as concrete while those near the bottom ranked as abstract. All comparisons with fMRI orders displayed positive linear relationships in the expected direction, with high statistical significance (Paradigm Class : Survey, 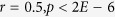; Behavioral Domain : Survey, 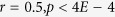; Neurosynth : Survey, 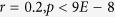).

One limitation of these studies is the interpretation of behavioral element order; the neuroscientific behavioral data are so complex, that for practical purposes their ranking must be treated as pseudo-subjective. Behavioral elements appear to sort via predicted theoretical constructs (abstraction), though must be judged manually, via intuition or experience (for an example, consider the lists in Fig. 6). Almost 500 participants sorted and judged our list of behavioral elements by whether the words seemed “concrete” versus “abstract” to them (Fig. 7).

Surveys produced rank orders, just as were produced by our physiological network-depth analyses. Order judged by human participants appeared to agreeably sort by “concreteness” versus “abstractness” confirming at least data quality and some consistency within a human sample’s intuition of the definition of “abstraction”, and that the participants did follow instructions on average.

Behavioral element orders generated via physiological network-depth from each behavioral database were compared to participants’ survey ordering, resulting in three sets of pairings: 1) Average-Neurosynth vs. Survey, 2) Average-Behavioral Domain vs. Survey, and 3) Average-Paradigm Class vs. Survey. Fascinatingly, the survey-derived orders and all fMRI-derived orders were positively correlated (highly statistically significant) for all database comparisons, quantifiably supporting the human interpretation of these results (Fig. 7). As expected, hand-coded and higher quality database comparisons, Behavioral Domain and Paradigm Class, showed a stronger relationship to the survey data than Neurosynth, which auto-extracts single words or phrases from full text articles.

Though some previous studies developed databases of “concreteness” ratings of words by surveying human participants for purposes primarily within linguistics or natural language processing, there is little to no overlap in content with BrainMap Behavioral Domain and Paradigm Class, and only moderate overlap with Neurosynth^52^,^53^. Where matching was possible, our survey results (neurosynth only) were generally congruent with previous norming studies^52^ in linguistics studying concreteness (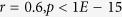).

This novel use of human participants ascertains whether results fit a pre-defined neuroscientific hypothesis pertaining to complex relationships regarding behavioral constructs which are cumbersome to quantify. Our hypothesized level of abstraction was validated via manual evaluation by surveying human participants.

### Validating methods and results

To verify these findings’ robustness, we repeated experiments with each behavioral database (Neurosynth, BrainMap), and both connectivity matrices separately (rsfMRI, DTI). Results replicated across independent behavioral databases, and across method of defining integrated connectivity (network mean, maximum). Binning the cognitive localization data (fMRI) by connectivity was performed for each modality and the integration of all modalities (8 connectivity sets), as well as for the meta-data categories BrainMap Behavioral Domain (Supplementary Data Table S3), BrainMap Paradigm class (Fig. 6a), and Neurosynth feature set (Fig. 6b, Supplementary Data Table S4).

To test the robustness of our findings, the results produced by each method of generating integrated connectivity (mean and max) along with each connectivity data type (rsfMRI and DTI) were compared with every other method’s resultant rank order of behavioral elements. To compare and validate rank orders between: DTI-Max, DTI-Mean, rsfMRI-Max, and rsfMRI-Mean, Pearson’s coefficient was computed for each pairwise comparison. All behavioral element results were strongly positively correlated as expected, with orders generated via DTI-Max and DTI-Mean showing the strongest relationship around (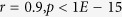) and DTI-Max and rsfMRI-Mean showing the weakest around (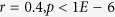), with full detail in Supplementary Data Tables S1. No matter what combination of method and cross-modality composite measures was used, our behavioral elements sorted similarly.

Our symbolic continuity hypothesis is most prominently illustrated by the lists of behavioral elements generated when binning the fMRI databases by the connectivity profile which integrated all inputs to cortex (Fig. 6a,b). However, each other result also supported the method and hypothesis. Notably, for internal quality control, the experiments were repeated for each modality separately, in addition to the integration of all modalities. The method validated itself, producing expected results for single modalities: e.g., for individual primary cortices, sensory tasks appeared at sensory ends of ranked lists. These myriad iterations all pointed to the same robust conclusions.

### Randomized and meaningful control experiments

**Figure 8:**
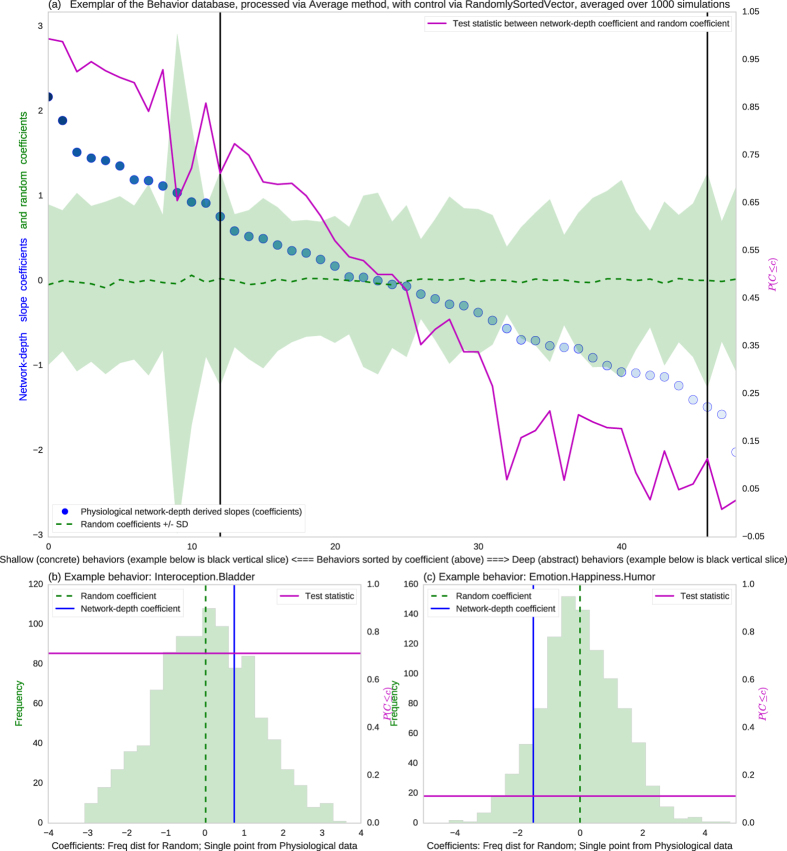
Randomized control sorting of behavioral elements. (**a**) Y-axis is slope (random and network-depth); X-axis indexes individual behaviors sorted by depth. Behavioral domain ranks were illustrated as one blue dot per slope and behavioral element, random distributions over 1000 simulations show mean in dashed green, with +/− SD as pale green, the probability of difference (1-tailed) plotted in magenta, and two exemplar behaviors marked by black vertical lines and illustrated in: (**b**,**c**) Each plot is a vertical slice of (**a**) corresponding to a black line above. Plots show non-random physiological network depth-slope (in blue) and the histogram of the random slopes in green.

To further validate findings, two random controls experiments were performed. Each control simulation processed data identically to the real experiment, except that connectivity vectors were randomized, either at the bin-ordering stage, or the connectivity vector generation stage (details in pseudo-code above). Notably, control simulations were identical to actual methods, except in the ordering of the connectivity vector for binning. Each random simulation was run 1000 times, with the entire distribution of results analyzed to generate test statistics. The sorting of behaviors by network-depth appeared non-random (Fig. 8). The variability of random controls was illustrated by the distribution of random coefficients and allowed comparing to the network-depth coefficients to produce a test statistic per behavioral element.

**Figure 9:**
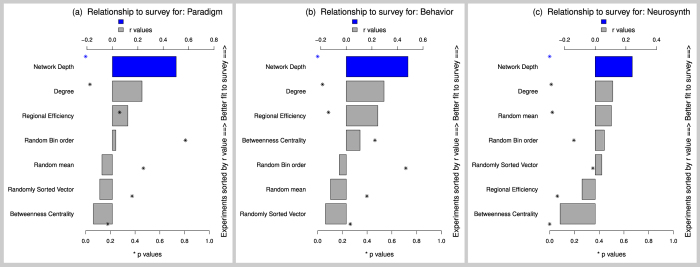
Network-depth orderings averaged across all experiments versus 5 control experiments (2 random and 3 meaningful controls). It is important to note that each experiment (names listed on Y-axis), was ordered by strength of relationship to the survey, with network-depth based sorts always the strongest, and thus at the top. (**a**) BrainMap Paradigm class, (**b**) BrainMap Behavioral domain), and (**c**) Neurosynth. As expected, higher quality hand-code BrainMap data produced stronger results than Neurosynth, which still showed all patterns in the expected direction. Experiments with 47 bins produced similar results (Supplementary Fig. S4).

Most importantly, two different random controls (randomized bins and randomized sorting vectors) as well as grand means of random controls sorted less well with the survey than did network-depth based sorts (Fig. 9). In addition to randomized control simulations, several meaningful controls were run, by sorting behavioral elements via other node-wise graph statistics, identically to sorts via network-depth. Survey ranks correlated more strongly with network-depth based sorting than it did with sorting via other graph statistics: betweenness centrality, degree, and regional efficiency (Fig. 9).

## Discussion

**Figure 10:**
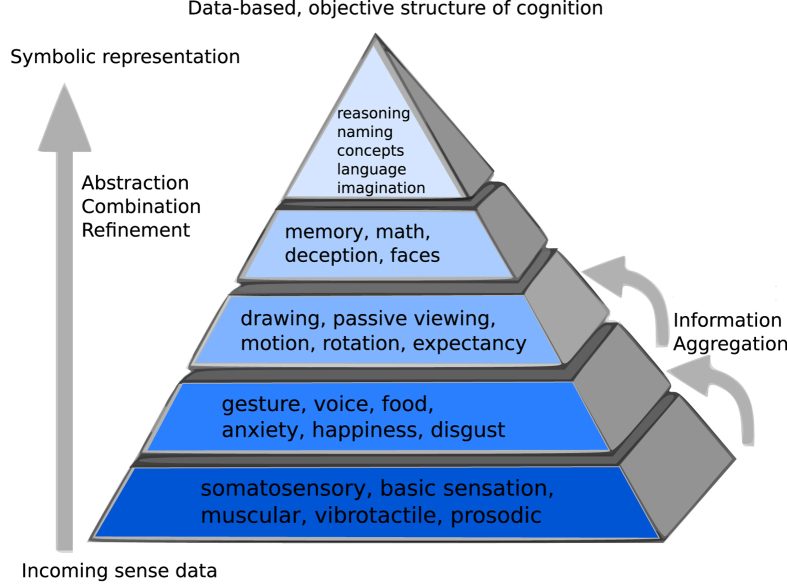
Data-based, objective pyramid of cognition. Generated by human-blind automated procedures, we depict an oversimplified graphical model of the information representation flow from sensory inputs (bottom) to abstract representations (top) in human cortex. Bottom layer of the pyramid included a sample representative description of the 20th percentile of behavioral elements closest to sensory inputs, the next layer up includes a sample description of behavioral elements from the 20–40th percentile…with the top layer containing a sample description of the behavioral elements distributed deepest in the cortical network, at the structural pinnacle of cognition.

Our symbolic continuity hypothesis appeared congruent with the order of ranked lists of behavioral elements derived from objective physiology and confirmed via human survey participants. Further, analyses replicated across different fMRI databases and data processing methods. These results agreed with the hypothesis that cortex displays global hierarchical organization of abstraction across regions, with simple features emerging near inputs to cortex, and with nodes having further connectivity-distance from inputs activated by greater aggregates of features, more predominately symbolic or complex functions, or more refined or abstract representations (Fig. 10).

As a caveat, the terms abstraction, aggregation, refinement, amalgamation, and combination are merely attempts to define the observed distribution of information processing which occurs in distributed neural network structures, though it is the unknown actuality of this general process which is of interest. For ranked lists, a cautious reader may conclude that sorting order was caused by one of many arbitrary features selected by our directed connectome model; e.g., with tactile functions observed at the sensory end, and auditory at the abstract end (Fig. 6). Several interesting dissociations suggest abstraction may indeed be at least one feature which associated with network distance from sensory input globally. At the sensory end of our lists, emerged tangible language-related behavioral elements such as: prosodic, speech execution, musical cognition, whistling, auditory discrimination, music comprehension/production, pitch, prosody, musical, tones, auditory perception. At the abstract end emerged features which at face-value appear similar to the preceding list, though perhaps vary in degree of symbolic content: naming, braille reading, word generation, word stem completion, phonological discrimination, phonology, readers, orthographic, languages, lexical, semantic, names, identity. Such fascinating dissociations argue against simpler interpretations, and allude to the information processing principles in logic and computer science, where abstraction is defined as: *a process of creating general concepts or representations by emphasizing common features from specific instances, where unified concepts are derived from literal, real, concrete, or tangible concepts, observations, or first principles, often with the goal of compressing the information content of a concept or an observable event, and retaining only information which is relevant for an individualized goal or action.* Though abstraction may vary with network-depth, it is also likely that other constructs may additionally explain the localization and processing of information illustrated by these directed cortical networks.

Past rsfMRI and DTI-based connectome models envisioned a network-based perspective; studies suggested that regionalization of cognitive function is related to the modularity, centrality, and network properties of nodes (regions)^54^,^55^,^56^. Cortical sub-networks can be isolated from single physiological rsfMRI studies, the largest sub-networks of which often include: motor-network, visual-network, extra-striate-visual, insular-temporal/anterior-cingulate-cortex, left-frontal-parietal, right-frontal-parietal, default-mode-network, and a frontal-network. Quite notably, similar major sub-networks^57^ or neighborhoods^58^ emerge when isolated using parallel methods, but instead applied to task-fMRI from thousands of studies^58^,^59^,^60^. Earlier findings confirm the congruence between connectivity and representation in the human cortex and validate both meta-task-fMRI databases and physiological connectivity methods. Using traditional single-study hypothesis testing, models of relationships between connectivity and representation in cortical networks were formed based on patching together the literature; notably, these past scientist-generated models tend to agree with objective systematic methods of evaluating the gross, global, or entire-community properties of brain connectivity networks^58^,^59^. Though structure and function of cortical networks, including hubs, neighborhoods, network efficiency, integrity, and hierarchical clustering^59^,^61^ have been described, past analyses did not specify gross directionality via input location, a critical feature for defining hierarchy or localization of representation within any neural network. We remedied this dearth by introducing a reliable method of adding global directionality to a cortical network model, enabling the analysis of functional hierarchical aggregation. Further, we contributed a method for associating geometrical distributions of behavioral fMRI activations in relation to any regional node-wise graph statistics derived from connectome data.

Our current data-driven results holistically demonstrated expanding amalgamation of function over structural depth, and also possess explanatory power for cognition, providing insights regarding the neuronal process of abstraction. Fascinatingly, results suggest why the insular and opercular regions are some of the best candidates for awareness or consciousness^62^,^63^, or why parts of frontal cortex are involved in higher cognition. These conclusions are elaborated here:

Sensory regions closest to inputs (*bin*_1_) in our hierarchy derived from the average of all senses in parallel included: insula, operculum, and immediately underlying claustrum. These regions have been hypothesized to play a role in multi-sensory integration and interoceptive awareness, and to be among the best regional correlates of consciousness^40^,^62^,^63^,^64^,^65^. It has been demonstrated that electrical stimulation of the human left claustrum/anterior insula during neurosurgery can arrest consciousness^66^. Notably, these findings complement predominant models of consciousness describing global integration^67^. These findings may explain the reason that the insular cortex is known to play a role in multi-sensory processing, since it is the hub of the senses. In conclusion, this may point to the coordinating role awareness plays in cognition^68^. With a presumed seat of consciousness or awareness positioned structurally in a location that would allow it to be a coordinator of sensory inputs and cognition, these studies may change our assumptions about consciousness and the design of machine learning and artificial intelligence. Further, middle regions, intermediate in distance from all sensory inputs (*bin*_5_) included hippocampus and MTL known for memory and celebrity neurons^69^. Deep regions less connected to and farthest from inputs (*bin*_10_) included: bilateral inferior temporooccipital, bilateral frontal poles, left cingulate anterior, and left inferior frontal pars triangularis, which may support more abstract cognitive functions.

From the word-orders derived from network-depth, displaying a linear relationship with participant-judged abstraction, one could hypothesize that the cognitive process of abstraction or symbol creation may be estimated by a relatively continuous and simple increase in symbolic content over network-depth, as is observed in computational networks. Deep artificial networks with many layers are known to naturally represent higher abstractions in deeper levels, more distant from inputs. We suggest that in this way, many artificial architectures are roughly similar to the brain, perhaps describing how both may construct symbols. These concepts agree with studies of semantics and linguistic symbol creation in neuroscience, which suggest that the neuronal instantiations of abstract words may be more variable than those of concrete words, particularly with abstract semantic circuits thought to be multimodal, prefrontal, and parietal regions^70^, which were some of the deepest regions in our network. Further, studies in psychology suggest that concrete words may be more accessible or memorable than abstract words, for example, producing slower reaction or decision times than to abstract words^71^. Such models would be congruent with the proposed neuronal coding in our study and others^70^.

In conclusion, we provided premier complete data-intensive experiments demonstrating symbolic continuity for grouped sensory modalities and cortex globally. The studies presented here descriptively illustrated, based on big neuroinformatics, that network-depth via connectivity-distance from sensory inputs predicted the distribution of abstraction of cognitive functions across regions globally, to frontal, parietal, temporal, and association cortices. This suggested a fundamental motif or organizational principle of brains. From tangible sensory inputs, symbolic content may progressively emerge as information is processed deeper into the brain’s structural network, starting with inputs, and expanding in abstraction or refinement, resulting in intangible or deep symbolic content in the structural pinnacle of the human brain network. These experiments successfully addressed a proverbial “elephant in the room” an assumption about brain function, sometimes taken as axiomatic, though at times objected, that seems simultaneously too grand, too simplistic, and too complicated to demonstrate comprehensively.

## Additional Information

**How to cite this article**: Taylor, P. *et al.* The global landscape of cognition: hierarchical aggregation as an organizational principle of human cortical networks and functions. *Sci. Rep.* **5**, 18112; doi: 10.1038/srep18112 (2015).

## Supplementary Material

Supplementary Information
