# Supplementary material for: The global landscape of cognition: hierarchical aggregation as an organizational principle of human cortical networks and functions
Source: Sci Rep. 2015 Dec 16;5:18112. doi: 10.1038/srep18112 (PMC4681187; doi:10.1038/srep18112)
Supplement: Supplementary Information [file srep18112-s1.pdf]

The global landscape of cognition: hierarchical aggregation as an organizational principle of human cortical networks and functions

P. Taylor[1,2,*], J.N. Hobbs[1], J. Burroni[1] and H.T. Siegelmann[1,2]

[1]College of Information and Computer Sciences
[2]Neuroscience and Behavior Program
University of Massachusetts
Amherst, MA, USA
[*]Corresponding author: ptaylor@cs.umass.edu

List of Supplementary Materials

- Fig. S1. Network-depth plotted over the maximum spanning tree
- Fig. S2. Behavioral activation counts plotted over the maximum spanning tree
- Fig. S3. Random simulation for Paradigm class
- Fig. S4. Random simulation for Neurosynth
- Table S1. Matrix of statistical comparisons between methods of processing
- Fig. S5. The effect of binning illustrated with 47 bins
- Fig. S6. Survey instructions given to human participants
- Table S2. Behavioral element rank orders for BrainMap Paradigm Class
- Table S3. Behavioral element rank orders for BrainMap Behavioral Domain
- Table S4. Behavioral element rank orders for Neurosynth

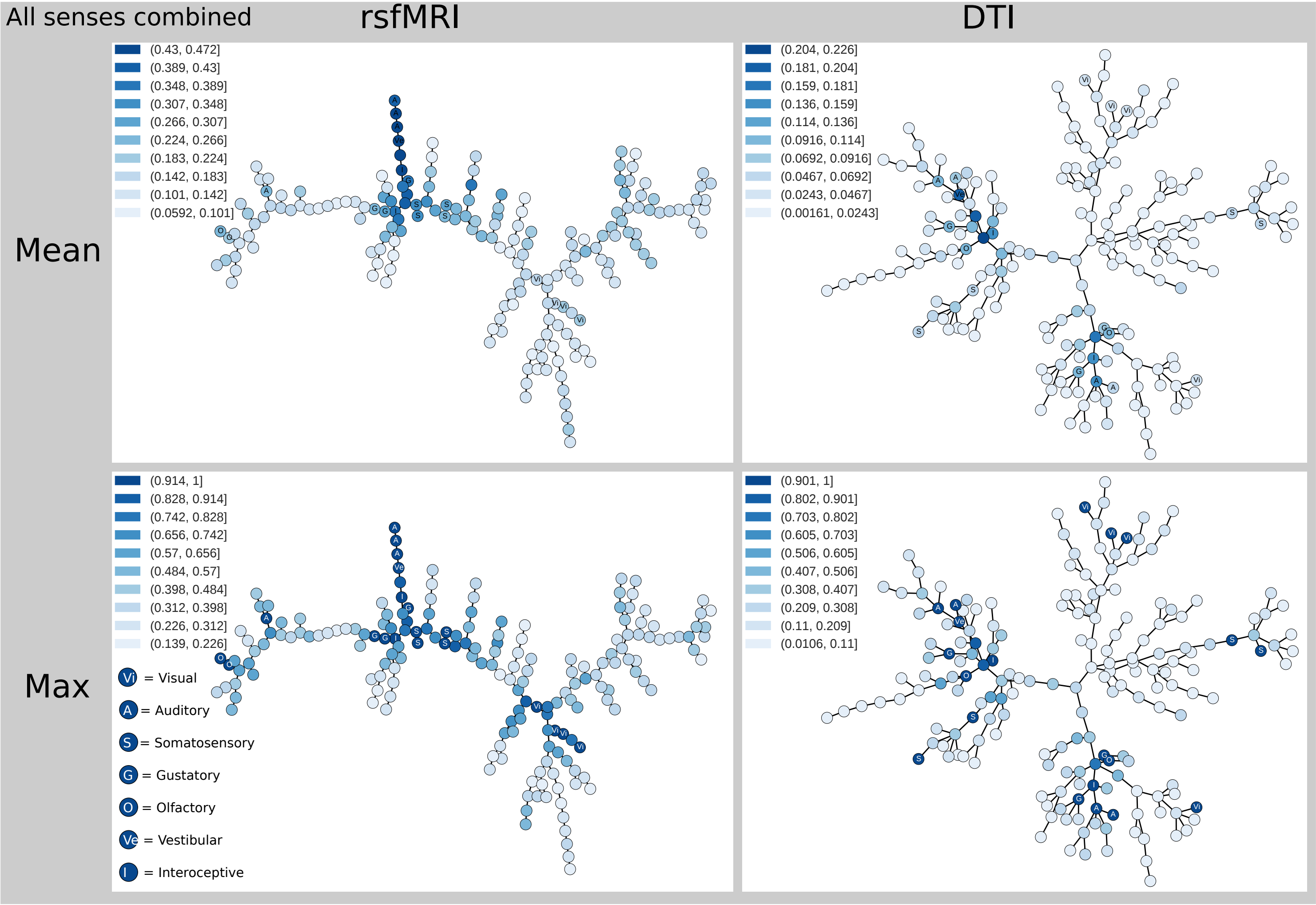

Figure S1: Network-depth via integrated sensory inputs displayed over whole-cortex, represented on nodes of the maximum spanning tree (MST), where darker blue is more connected to inputs. Input nodes were marked by labels described in bottom left legend.

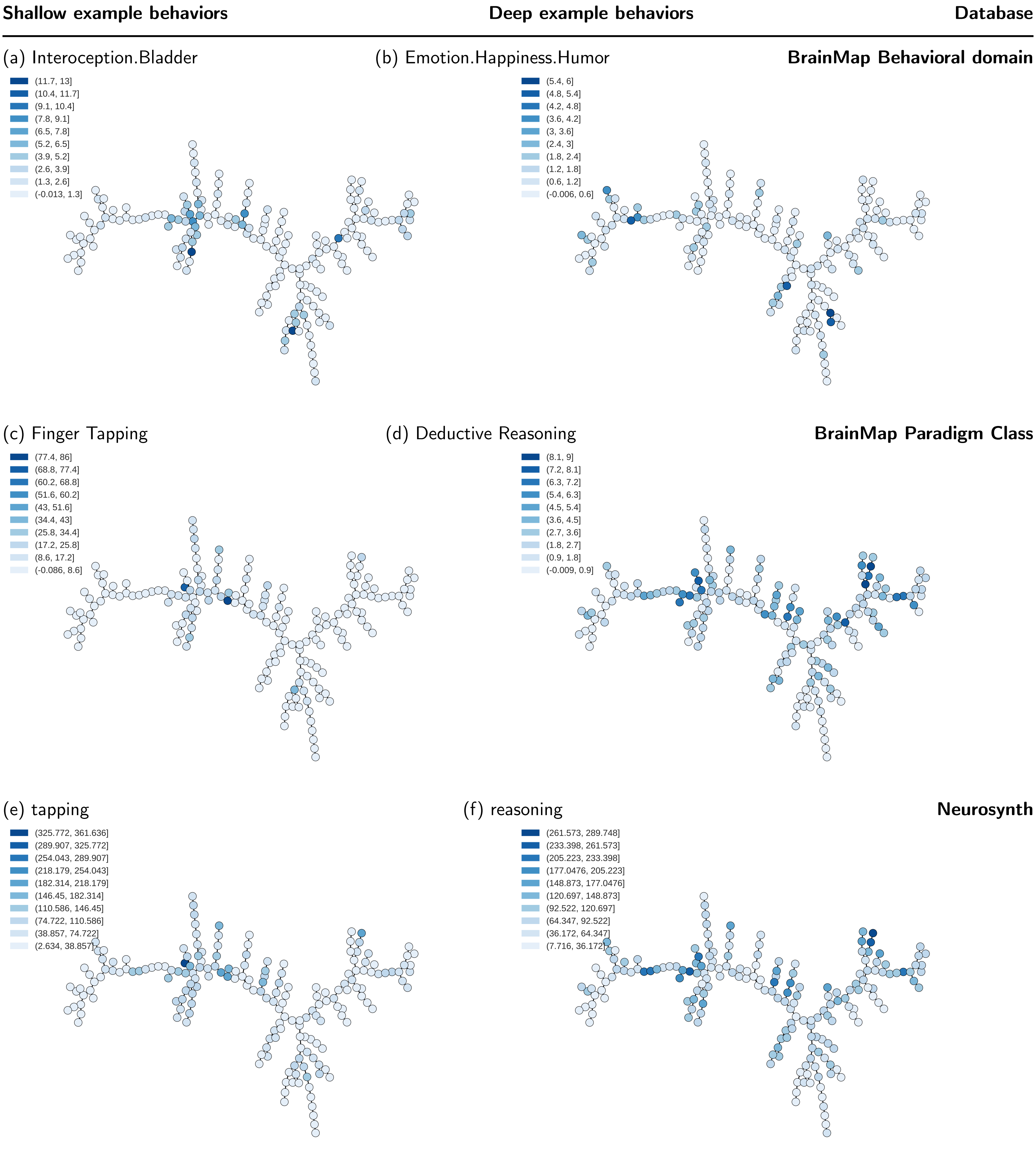

Figure S2: MST for example behaviors, with darker blue indicating higher activation counts per node. **(a-f)** One shallow (left) and deep (right) behavior per database. Abstract behavioral elements tended to be distributed more broadly, populating several branches and multiple nodes. On the other hand, sensory behavioral elements tended to cluster around sensory regions.

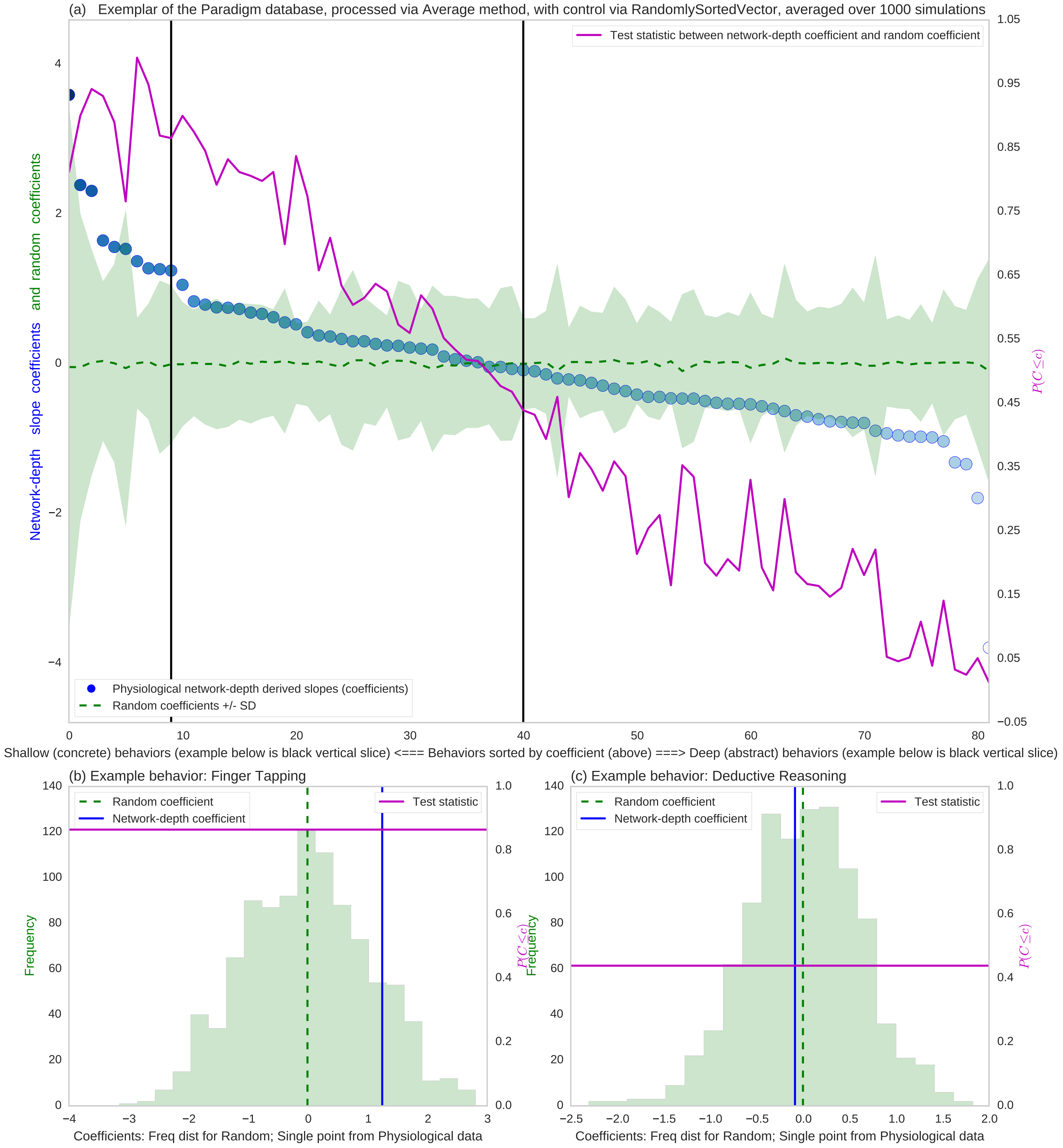

Figure S3: Randomized control sorting of behavioral elements for BrainMap Paradigm Class. **(a)** Y-axis is slope (random and network-depth); X-axis indexes individual behaviors sorted by depth. Behavioral domain ranks were illustrated as one blue dot per slope and behavioral element, random distributions over 1000 simulations show mean in dashed green, with +/- SD as pale green, the probability of difference (1-tailed) plotted in magenta, and two exemplar behaviors marked by black vertical lines and illustrated in: **(b,c)** Each plot is a vertical slice of **(a)** corresponding to a black line above. Plots show non-random physiological network depth-slope (in blue) and the histogram of the random slopes in green.

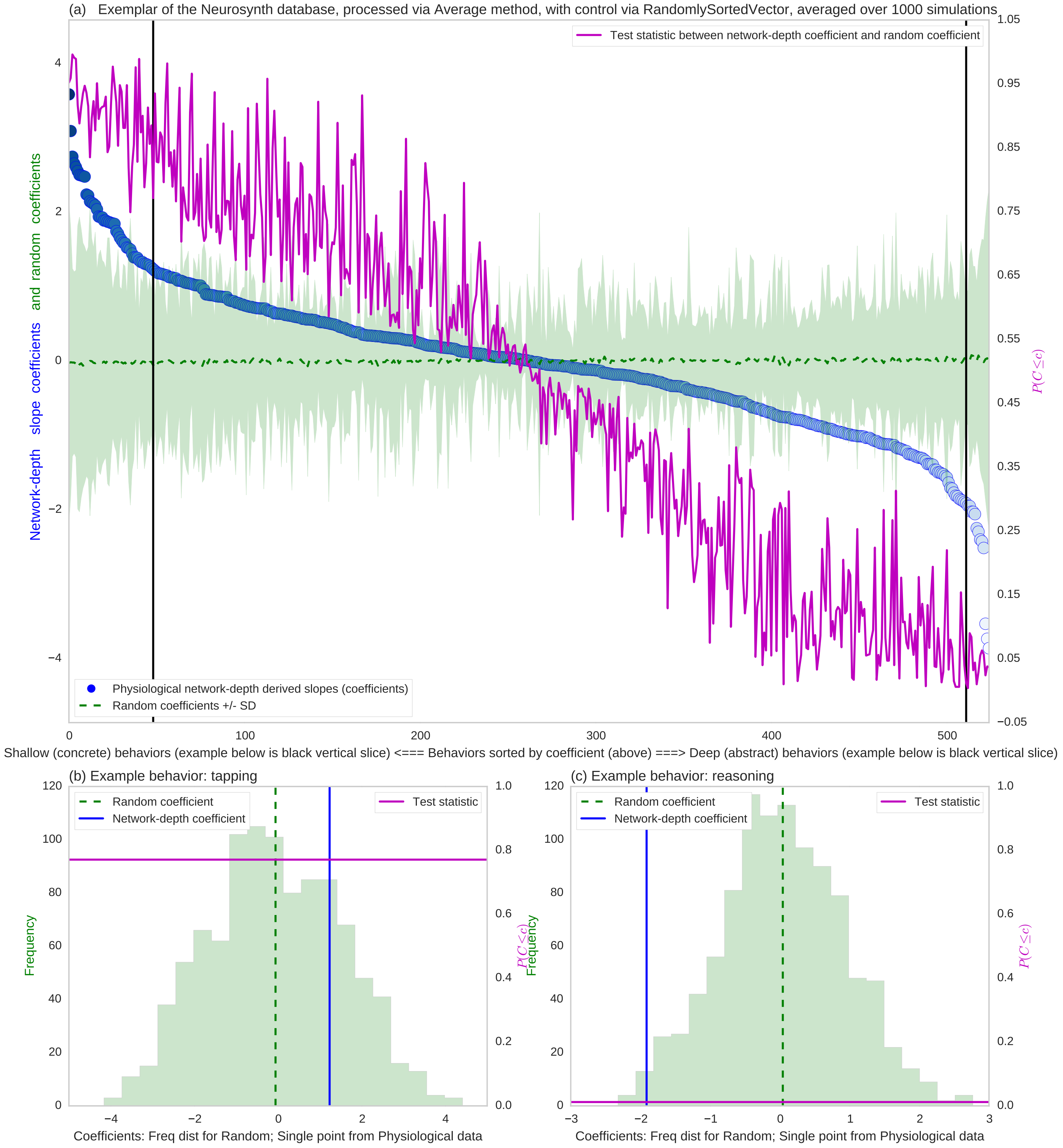

Figure S4: Randomized control sorting of behavioral elements for the Neurosynth database. Details as in Fig. S3.

(a) BrainMap: Behavioral Domain

			**p values**
DTI Average	<1.0E-15	4.19464171739214E-005	2.12783124453608E-010
0.94255331	DTI Max	0.0080164283	1.52029113742458E-008
0.5503145932	0.3745406196	rsfMRI Average	3.40330874593064E-010
0.761419631	0.705384928	0.7559104523	rsfMRI Max
**r values**			

(b) BrainMap: Paradigm Class

			**p values**
DTI Average	<1.0E-15	3.60205874239128E-007	0.00000001
0.7982462931	DTI Max	2.81255954637238E-006	8.43769498715119E-015
0.5272235695	0.4910570554	rsfMRI Average	4.66581795421916E-009
0.5816090613	0.7287044136	0.5920800617	rsfMRI Max
**r values**			

(b) Neurosynth

			**p values**
DTI Average	<1.0E-15	<1.0E-15	<1.0E-15
0.8699921649	DTI Max	<1.0E-15	<1.0E-15
0.6517654055	0.408950743	rsfMRI Average	<1.0E-15
0.752321367	0.771785629	0.7607496363	rsfMRI Max
**r values**			

Table S1: Correlation between methods for generating connectivity to sort behavioral element rank orders (DTI Average, DTI Max, rsfMRI Average, and rsfMRI Max). Table of pairwise comparisons using Pearson's product moment correlation coefficient. Intersections between diagonal labels tabulate comparisons, with bottom matrix intersection displaying *r* values and top displaying *p* values; *p* values $< 1.0E-15$ rounding to 0 displayed as $< 1.0E-15$. **(a)** Behavioral Domain. **(b)** Paradigm Class, **(c)** Neurosynth. Each method demonstrates high degree of consistency in generating rank orders of behavioral elements.

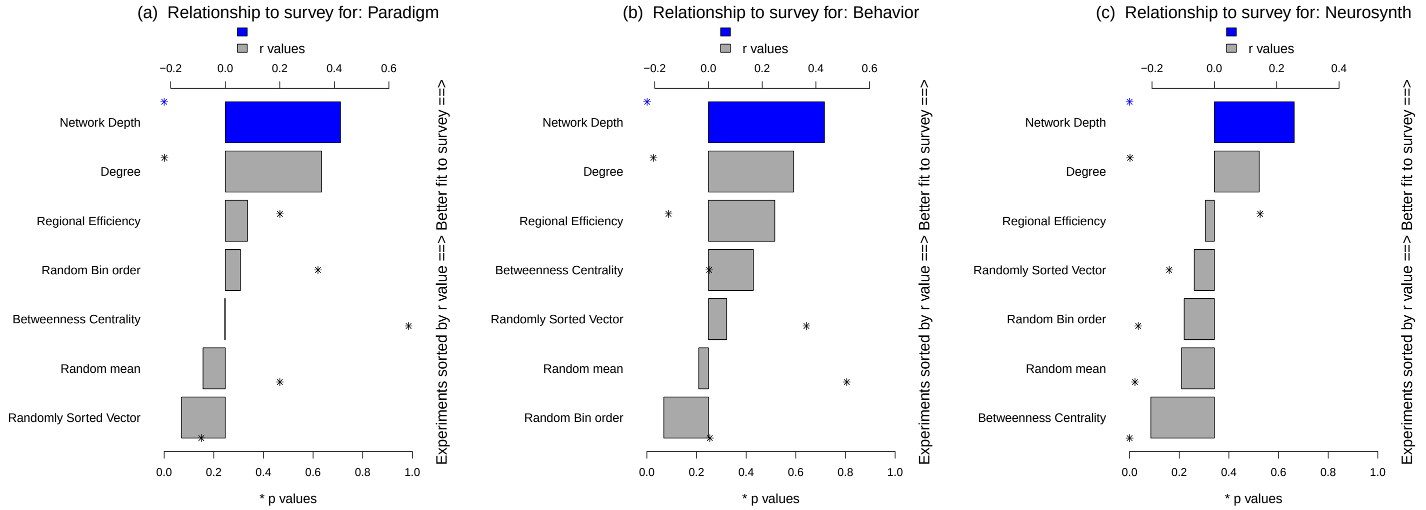

Figure S5: Network-depth orderings with 47 bins averaged across all experiments versus 5 control experiments (2 random and 3 meaningful controls). It is important to note that each experiment (names listed on Y-axis), was ordered by strength of relationship to the survey, with network-depth based sorts always the strongest, and thus at the top. **(a)** BrainMap Paradigm class, **(b)** BrainMap Behavioral domain), and **(c)** Neurosynth.

(a) Survey instructions:

We need your help sorting neuroscience phrases by how “Abstract” or “Concrete" they are.

CONCRETE is defined as:
* a real thing or class of things;
* solid; definite objects or events that are available to the senses;
* Examples include: your chair, tickling, seeing brown, touching rough, a soccer ball, a particular house, your favorite song
* capable of being touched;
* pertaining to or representing an actual thing or specific instances, rather than abstractions, or abstract qualities
* particular (opposed to general);
* characterized by immediate experience of actual things or events;
* existing in real experience, material or physical form;
* substantial; not vague or elusive;
* actual, rather than imaginary or visionary;
* palpable; tangible, simple, sensory.

ABSTRACTION is defined as:
* a process of creating general concepts or representations by emphasizing common features from specific instances, where unified concepts are derived from literal, real, concrete, or tangible concepts, observations, or first principles, often with the goal of compressing the information content of a concept or an observable event, and retaining only information which is relevant for an individualized goal or action;
* Process of information aggregation, refinement, combination, integration, coalescing, accumulation, amalgamation, combination of ideas;
* In mathematics: process of extracting the underlying essence of a mathematical concept, removing any dependence on real world objects with which it might originally have been connected, and generalizing it so that it has wider applications;
* In computer science: process of separating ideas from specific instances of those ideas at work;
* In linguistics: process in the development of language, whereby terms become used for concepts further removed from the objects to which they were originally attached;

ABSTRACT is defined as:
* Existing in thought, concept, or idea, but not having a physical or concrete existence;
* A general idea or quality rather than an actual person, object, or event;
* not easy to understand because of being extremely complex, remote from concrete reality;
* expressing a quality or characteristic disassociated from any concrete reality, specific object, actual instance, material basis, specific material object, or particular instance;
* Examples include: justice, truth, poverty, beauty, and speed;
* A concept that acts as a super-categorical noun for all subordinate concepts, and connects any related concepts as a group, field, or category;
* theoretical; not applied or practical; abstruse;
* dealing with ideas rather than events; theoretical;

Please help us sort the upcoming 1 page of neuroscience related phrases based on your judgment of whether they are CONCRETE versus ABSTRACT.

(b) Sorting question example:

Instructions: Sort the following phrases, activities, ideas, tasks, or concepts by how “Concrete” versus “Abstract” they seem, or would be to experience, by using the mouse to drag one phrase into each box.

Reset Cards

Emotion.Sadness
Interoception.Air-Hunger (feeling of hunger)
Cognition.Music
Emotion.Fear
Perception.Vision.Color (seeing basic colors)
Perception.Somesthesis.Pain (physical pain)
Perception.Vision

1 Most Concrete (least abstract) | 2 | 3
4 (neutral) | 5 | 6
7 Most Abstract (least concrete)

(c) Scale question example:

Finger Tapping

Instructions: Rank the above phrase, idea, activity, task, or concept by how “Concrete” versus “Abstract” it seems, or would be to experience.

1 Most Concrete (least abstract)	2	3	4 (neutral)	5	6	7 Most Abstract (least concrete)
○	○	○	○	○	○	○

Figure S6: Validation survey employing human participants. **(a)** Instructions provided to participants before survey. **(b)** Sample question requiring participants to use the mouse drag each phrase into a box for sorting behavioral elements. **(c)** Sample question requiring participants to rank a single behavioral element in isolation.

Paradigm Class

Table S2. Behavioral element rank orders (Paradigm Class).

Word list orders were generated by each connectivity source (DTI, rsfMRI) and integration method (Max, Average).

Abstract-distributed tasks tend to emerge near the top of the list, and sensory behaviors at the bottom of the list.

**DTI Average**	**DTI Max**	**rsfMRI Average**	**rsfMRI Max**	**Survey Average**
Braille Reading	Anti-Saccades	Naming (Covert)	Delay Discounting Task	Deductive Reasoning
n-back	Delay Discounting Task	Subjective Emotional Picture Discrimination	n-back	Semantic Monitor/Discrimination
Naming (Covert)	n-back	Reward Task	Anti-Saccades	Deception Task
Subjective Emotional Picture Discrimination	Saccades	Breath-Holding	Tower of London	Delay Discounting Task
Anti-Saccades	Video Games	Encoding	Reward Task	Go/No-Go
Delay Discounting Task	Mental Rotation	n-back	Word Stem Completion (Overt)	Imagined Objects/Scenes
Imagined Objects/Scenes	Imagined Objects/Scenes	Posner Task	Paired Associate Recall	Simon Task
Orthographic Discrimination	Orthographic Discrimination	Delay Discounting Task	Sternberg Task	Sequence Recall/Learning
Wisconsin Card Sorting Test	Pointing	Word Generation (Covert)	Imagined Objects/Scenes	Paired Associate Recall
Theory of Mind Task	Subjective Emotional Picture Discrimination	Stroop Task	Subjective Emotional Picture Discrimination	Syntactic Discrimination
Mental Rotation	Theory of Mind Task	Delayed Match To Sample	Stroop Task	Phonological Discrimination
Semantic Monitor/Discrimination	Braille Reading	Naming (Overt)	Task Switching	Cued Explicit Recognition
Pointing	Visual Pursuit/Tracking	Semantic Monitor/Discrimination	Delayed Match To Sample	Stroop Task
Reading (Covert)	Wisconsin Card Sorting Test	Braille Reading	Deception Task	Passive Viewing
Word Stem Completion (Covert)	Stroop Task	Phonological Discrimination	Wisconsin Card Sorting Test	Word Generation (Covert)
Word Generation (Overt)	Fixation	Tower of London	Word Generation (Covert)	n-back
Acupuncture	Deductive Reasoning	Word Stem Completion (Overt)	Cued Explicit Recognition	Theory of Mind Task
Visual Pursuit/Tracking	Word Stem Completion (Covert)	Paired Associate Recall	Counting/Calculation	Passive Listening
Naming (Overt)	Divided Auditory Attention	Action Observation	Word Generation (Overt)	Encoding
Task Switching	Acupuncture	Wisconsin Card Sorting Test	Naming (Covert)	Naming (Covert)
Saccades	Spatial/Location Discrimination	Word Generation (Overt)	Orthographic Discrimination	Oddball Discrimination
Cued Explicit Recognition	Flanker Task	Spatial/Location Discrimination	Video Games	Wisconsin Card Sorting Test
Deductive Reasoning	Episodic Recall	Task Switching	Mental Rotation	Imagined Movement
Drawing	Deception Task	Imagined Objects/Scenes	Encoding	Flanker Task
Action Observation	Visual Distractor/Visual Attention	Mental Rotation	Episodic Recall	Orthographic Discrimination
Paired Associate Recall	Go/No-Go	Sternberg Task	Theory of Mind Task	Posner Task
Counting/Calculation	Naming (Covert)	Counting/Calculation	Free Word List Recall	Episodic Recall
Spatial/Location Discrimination	Cued Explicit Recognition	Cued Explicit Recognition	Deductive Reasoning	Music Comprehension/Production
Divided Auditory Attention	Paired Associate Recall	Episodic Recall	Word Stem Completion (Covert)	Word Stem Completion (Covert)
Encoding	Writing	Reading (Covert)	Non-Painful Thermal Stimulation	Spatial/Location Discrimination
Writing	Semantic Monitor/Discrimination	Visual Distractor/Visual Attention	Saccades	Mental Rotation
Stroop Task	Task Switching	Deception Task	Spatial/Location Discrimination	Sternberg Task
Deception Task	Sternberg Task	Orthographic Discrimination	Semantic Monitor/Discrimination	Pain Monitor/Discrimination
Episodic Recall	Counting/Calculation	Film Viewing	Go/No-Go	Visual Distractor/Visual Attention
Delayed Match To Sample	Delayed Match To Sample	Passive Viewing	Film Viewing	Reading (Covert)
Passive Viewing	Encoding	Face Monitor/Discrimination	Phonological Discrimination	Subjective Emotional Picture Discrimination
Phonological Discrimination	Sequence Recall/Learning	Anti-Saccades	Visual Distractor/Visual Attention	Anti-Saccades
Go/No-Go	Free Word List Recall	Free Word List Recall	Rest	Task Switching
Video Games	Reading (Covert)	Go/No-Go	Posner Task	Classical Conditioning
Flanker Task	Passive Viewing	Pitch Monitor/Discrimination	Acupuncture	Recitation/Repetition (Covert)
Free Word List Recall	Word Generation (Overt)	Syntactic Discrimination	Flanker Task	Delayed Match To Sample
Word Generation (Covert)	Simon Task	Word Stem Completion (Covert)	Olfactory Monitor/Discrimination	Word Stem Completion (Overt)
Visual Distractor/Visual Attention	Naming (Overt)	Recitation/Repetition (Covert)	Syntactic Discrimination	Tower of London
Sternberg Task	Imagined Movement	Visual Pursuit/Tracking	Action Observation	Word Generation (Overt)
Word Stem Completion (Overt)	Action Observation	Micturition Task	Pointing	Olfactory Monitor/Discrimination

Paradigm Class

Fixation	Tower of London	Acupuncture	Micturition Task	Visual Pursuit/Tracking
Sequence Recall/Learning	Drawing	Deductive Reasoning	Reading (Covert)	Divided Auditory Attention
Simon Task	Micturition Task	Theory of Mind Task	Fixation	Flashing Checkerboard
Imagined Movement	Rest	Olfactory Monitor/Discrimination	Recitation/Repetition (Covert)	Free Word List Recall
Film Viewing	Isometric Force	Rest	Passive Viewing	Reward Task
Grasping	Non-Painful Thermal Stimulation	Eating/Drinking	Drawing	Transcranial Magnetic Stimulation
Rest	Grasping	Fixation	Divided Auditory Attention	Face Monitor/Discrimination
Tower of London	Word Generation (Covert)	Recitation/Repetition (Overt)	Visual Pursuit/Tracking	Pitch Monitor/Discrimination
Face Monitor/Discrimination	Phonological Discrimination	Flanker Task	Naming (Overt)	Tone Monitor/Discrimination
Recitation/Repetition (Covert)	Word Stem Completion (Overt)	Non-Painful Thermal Stimulation	Sequence Recall/Learning	Writing
Micturition Task	Film Viewing	Saccades	Isometric Force	Vibrotactile Monitor/Discrimination
Non-Painful Thermal Stimulation	Reward Task	Drawing	Imagined Movement	Naming (Overt)
Flashing Checkerboard	Tactile Monitor/Discrimination	Simon Task	Braille Reading	Non-Painful Electrical Stimulation
Isometric Force	Posner Task	Writing	Writing	Fixation
Tactile Monitor/Discrimination	Pain Monitor/Discrimination	Tone Monitor/Discrimination	Classical Conditioning	Action Observation
Tone Monitor/Discrimination	Transcranial Magnetic Stimulation	Classical Conditioning	Tone Monitor/Discrimination	Drawing
Eating/Drinking	Tone Monitor/Discrimination	Sequence Recall/Learning	Face Monitor/Discrimination	Rest
Transcranial Magnetic Stimulation	Face Monitor/Discrimination	Reading (Overt)	Simon Task	Counting/Calculation
Reward Task	Recitation/Repetition (Covert)	Pain Monitor/Discrimination	Eating/Drinking	Saccades
Posner Task	Finger Tapping	Pointing	Pain Monitor/Discrimination	Micturition Task
Classical Conditioning	Classical Conditioning	Divided Auditory Attention	Recitation/Repetition (Overt)	Isometric Force
Pain Monitor/Discrimination	Chewing/Swallowing	Transcranial Magnetic Stimulation	Pitch Monitor/Discrimination	Film Viewing
Flexion/Extension	Olfactory Monitor/Discrimination	Passive Listening	Transcranial Magnetic Stimulation	Non-Painful Thermal Stimulation
Passive Listening	Flexion/Extension	Imagined Movement	Tactile Monitor/Discrimination	Breath-Holding
Finger Tapping	Eating/Drinking	Oddball Discrimination	Music Comprehension/Production	Reading (Overt)
Music Comprehension/Production	Music Comprehension/Production	Isometric Force	Passive Listening	Tactile Monitor/Discrimination
Syntactic Discrimination	Passive Listening	Music Comprehension/Production	Grasping	Acupuncture
Whistling	Recitation/Repetition (Overt)	Flexion/Extension	Oddball Discrimination	Recitation/Repetition (Overt)
Recitation/Repetition (Overt)	Whistling	Whistling	Finger Tapping	Pointing
Olfactory Monitor/Discrimination	Oddball Discrimination	Finger Tapping	Flexion/Extension	Braille Reading
Reading (Overt)	Reading (Overt)	Tactile Monitor/Discrimination	Whistling	Whistling
Chewing/Swallowing	Breath-Holding	Chewing/Swallowing	Reading (Overt)	Video Games
Breath-Holding	Syntactic Discrimination	Grasping	Chewing/Swallowing	Flexion/Extension
Oddball Discrimination	Vibrotactile Monitor/Discrimination	Vibrotactile Monitor/Discrimination	Non-Painful Electrical Stimulation	Eating/Drinking
Vibrotactile Monitor/Discrimination	Pitch Monitor/Discrimination	Video Games	Flashing Checkerboard	Grasping
Pitch Monitor/Discrimination	Flashing Checkerboard	Flashing Checkerboard	Vibrotactile Monitor/Discrimination	Finger Tapping
Non-Painful Electrical Stimulation	Non-Painful Electrical Stimulation	Non-Painful Electrical Stimulation	Breath-Holding	Chewing/Swallowing

Behavioral Domain

Table S3. Behavioral element rank orders (Behavioral Domain).

Word list orders were generated by each connectivity source (DTI, rsfMRI) and integration method (Max, Average).

Abstract-distributed tasks tend to emerge near the top of the list, and sensory behaviors at the bottom of the list.

DTI Average	DTI Max	rsfMRI Average	rsfMRI Max	Survey Average
Cognition.Language.Orthography	Action.Inhibition	Perception.Vision.Color	Cognition.Memory.Working	Action.Imagination
Cognition.Memory.Working	Perception.Vision.Motion	Emotion.Happiness.Humor	Cognition.Memory	Cognition.Reasoning
Cognition.Memory	Cognition.Memory.Working	Cognition.Language.Semantics	Perception.Vision.Color	Emotion.Fear
Action.Inhibition	Cognition.Space	Perception.Vision.Shape	Cognition.Reasoning	Cognition
Cognition.Social Cognition	Cognition.Memory	Cognition.Language.Phonology	Cognition.Social Cognition	Cognition.Space
Cognition.Space	Perception.Vision	Cognition.Memory	Cognition.Memory.Explicit	Interoception.Sexuality
Cognition.Language.Semantics	Cognition.Social Cognition	Cognition.Memory.Working	Action.Inhibition	Cognition.Memory.Working
Perception.Vision.Shape	Cognition.Language.Orthography	Action.Observation	Cognition.Space	Cognition.Language.Orthography
Emotion.Happiness.Humor	Cognition.Reasoning	Cognition.Language.Orthography	Cognition	Emotion.Anger
Perception.Vision	Cognition.Memory.Explicit	Cognition.Language.Syntax	Cognition.Language.Phonology	Cognition.Language
Cognition.Reasoning	Perception.Vision.Shape	Interoception.Air-Hunger	Emotion.Happiness.Humor	Action.Inhibition
Cognition.Memory.Explicit	Emotion.Happiness.Humor	Cognition.Space	Cognition.Language.Semantics	Cognition.Language.Semantics
Perception.Vision.Motion	Cognition.Attention	Cognition.Memory.Explicit	Action.Observation	Emotion
Cognition	Action.Imagination	Emotion.Anger	Emotion	Cognition.Social Cognition
Cognition.Language.Phonology	Action.Preparation	Perception.Vision	Cognition.Time	Cognition.Soma
Cognition.Time	Cognition	Cognition	Action.Rest	Cognition.Memory.Explicit
Cognition.Language	Cognition.Language.Semantics	Emotion	Interoception.Air-Hunger	Emotion.Disgust
Action.Observation	Interoception.Air-Hunger	Emotion.Fear	Cognition.Language.Syntax	Emotion.Happiness.Humor
Action.Imagination	Interoception.Bladder	Cognition.Language	Perception.Vision.Motion	Perception.Somesthesis
Interoception.Hunger	Action.Motor Learning	Cognition.Reasoning	Emotion.Sadness	Cognition.Attention
Cognition.Attention	Cognition.Time	Interoception.Hunger	Cognition.Attention	Emotion.Sadness
Interoception.Air-Hunger	Interoception.Thirst	Interoception.Sexuality	Cognition.Language.Orthography	Emotion.Happiness
Perception.Vision.Color	Action.Observation	Emotion.Sadness	Emotion.Anxiety	Perception.Vision
Action.Motor Learning	Perception.Vision.Color	Interoception.Thirst	Interoception.Hunger	Interoception.Hunger
Interoception.Bladder	Action.Rest	Cognition.Social Cognition	Emotion.Anger	Cognition.Memory
Action.Preparation	Emotion.Anxiety	Emotion.Anxiety	Perception.Olfaction	Cognition.Music
Interoception.Sexuality	Interoception.Hunger	Action.Inhibition	Perception.Vision.Shape	Interoception.Air-Hunger
Interoception.Thirst	Interoception.Sexuality	Cognition.Time	Interoception.Bladder	Action.Preparation
Emotion	Cognition.Language.Phonology	Cognition.Attention	Perception.Vision	Cognition.Language.Syntax
Action.Rest	Cognition.Language	Interoception.Thermoregulation	Interoception.Sexuality	Cognition.Time
Emotion.Anger	Emotion	Emotion.Disgust	Cognition.Language	Emotion.Anxiety
Cognition.Language.Speech	Cognition.Soma	Cognition.Language.Speech	Action.Preparation	Perception.Vision.Color
Emotion.Sadness	Emotion.Sadness	Emotion.Happiness	Action.Motor Learning	Perception.Gustation
Emotion.Anxiety	Perception.Somesthesis.Pain	Perception.Gustation	Perception.Gustation	Action.Observation
Emotion.Happiness	Emotion.Happiness	Action.Rest	Emotion.Fear	Action.Rest
Emotion.Fear	Emotion.Fear	Interoception.Bladder	Action.Imagination	Cognition.Language.Speech
Cognition.Language.Syntax	Perception.Somesthesis	Perception.Olfaction	Emotion.Happiness	Perception.Vision.Motion
Cognition.Soma	Action.Execution	Perception.Vision.Motion	Emotion.Disgust	Interoception.Bladder
Emotion.Disgust	Emotion.Anger	Interoception.Sleep	Interoception.Thirst	Interoception.Thermoregulation
Action.Execution	Cognition.Language.Speech	Action.Preparation	Cognition.Language.Speech	Perception.Vision.Shape
Interoception.Sleep	Emotion.Disgust	Action.Motor Learning	Perception.Somesthesis.Pain	Perception.Olfaction
Perception.Gustation	Perception.Olfaction	Cognition.Soma	Cognition.Soma	Cognition.Language.Phonology
Perception.Somesthesis	Interoception.Sleep	Perception.Somesthesis.Pain	Interoception.Thermoregulation	Perception.Audition
Perception.Somesthesis.Pain	Perception.Gustation	Perception.Audition	Cognition.Music	Action.Motor Learning
Action.Execution.Speech	Cognition.Music	Action.Execution.Speech	Perception.Somesthesis	Action.Execution.Speech

Behavioral Domain

Cognition.Music	Action.Execution.Speech	Action.Imagination	Perception.Audition	Interoception.Sleep
Perception.Olfaction	Cognition.Language.Syntax	Cognition.Music	Interoception.Sleep	Interoception.Thirst
Perception.Audition	Perception.Audition	Perception.Somesthesis	Action.Execution.Speech	Perception.Somesthesis.Pain
Interoception.Thermoregulation	Interoception.Thermoregulation	Action.Execution	Action.Execution	Action.Execution

Table S4. Behavioral element rank orders (Neurosynth)

Word list orders were generated by each connectivity source (DTI, rsfMRI) and integration method (Max, Average).

Abstract-distributed tasks tend to emerge near the top of the list, and sensory behaviors at the bottom of the list.

DTI Average	DTI Max	rsfMRI Average	rsfMRI Max	Survey Average
moral	selfreferential	phonology	2back	motivation
selfreferential	causality	readers	reasoning	anxiety
reasoning	moral	orthographic	wm	recall
stories	solving	languages	cards	identity
mentalizing	reasoning	letter	win	virtual
causality	tom	famous	solving	feelings
solving	mentalizing	relational	game	conditioning
famous	errorrelated	lexical	impulsivity	success
tom	thoughts	semantically	money	concepts
decisions	thinking	naming	arithmetic	inhibition
story	game	semantic	relational	conceptual
2back	2back	nouns	decisions	theory
readers	stroop	concepts	causality	meaning
thoughts	decisions	decisions	concepts	emotionally
thinking	saccade	phonological	moral	affect
stroop	wm	photographs	thinking	empathic
judgments	stories	remembered	1back	memories
game	executive	pseudowords	rewards	covert
intentions	autobiographical	nonwords	episodic	personality
errorrelated	traits	letters	nback	past
verb	shifting	names	digits	congruency
name	intentions	reasoning	remembered	judgments
letter	attribution	reading	dopaminergic	incongruent
executive	adolescence	verb	tom	unfamiliar
intentional	famous	1back	reinforcement	strategy
person	visuospatial	unfamiliar	visuospatial	intentions
congruency	perspective	decision	executive	perspective
letters	cards	scenes	letter	empathy
traits	arithmetic	identity	shifting	difficulty
conceptual	decisionmaking	animals	decisionmaking	judgment
attribution	impulsivity	word	incentive	familiar
concepts	maintenance	name	maintenance	tom
autobiographical	episodic	incentive	working	implicit
reading	outcomes	win	remember	endogenous
names	intentional	impulsivity	retrieval	affective
personality	trait	ambiguity	memories	decisionmaking
shifting	personality	episodic	retrieved	spatially
retrieval	judgments	2back	losses	congruent
adolescence	sleep	retrieved	success	moral
objects	remembered	scene	addiction	preference
semantic	card	syntactic	stroop	perception
remembered	imagine	remember	famous	stroop
unfamiliar	monitoring	distractors	outcomes	perceptual
shapes	saccades	category	reward	nback
perspective	story	judgment	digit	novelty

nonverbal	person	faces	letters	emotion
visuospatial	mental	retrieval	nouns	engaged
arithmetic	past	recollection	incorrect	time
identity	imagery	recall	noun	categorical
judgment	saccadic	spoken	decision	character
lexical	nback	english	recall	pleasant
episodic	concepts	shapes	autobiographical	knowledge
distractors	cued	noun	monetary	thinking
past	imagined	objects	recollection	developmental
prime	self	categorization	eating	causality
recollection	1back	visuospatial	episode	probabilities
1back	tracking	topdown	intelligence	remember
word	retrieval	intelligence	retention	episodic
maintenance	social	recognition	character	remembered
wm	reversal	pictures	encoded	famous
autistic	distractors	familiarity	stories	executive
social	conflict	memories	rules	categorization
persons	personal	encoded	distractors	schizophrenic
scenarios	conceptual	retention	nonverbal	familiarity
character	congruency	judgments	story	autistic
semantically	remember	read	verb	learned
pictures	character	colour	demand	conscious
incongruent	memories	strategies	judgments	loss
scene	prime	meaning	risk	comprehension
trait	scenarios	stroop	conceptual	experiences
memories	orienting	wm	selfreferential	mental
decision	ability	success	readers	unpleasantness
saccade	letter	conceptual	demands	ambiguous
competition	recollection	demands	longterm	salience
remember	effort	language	serial	social
engagement	persons	rewards	errorrelated	intelligence
decisionmaking	demands	priming	thoughts	choice
naming	scene	linguistic	judgment	uncertainty
mental	intention	incorrect	card	imagine
reversal	letters	shifting	smoking	imagery
demands	search	monetary	rule	semantically
cards	engagement	digits	load	angry
search	eye	writing	numerical	improvement
contexts	virtual	strategy	strategies	spatial
picture	competition	pairs	text	control
imagery	shapes	character	semantic	valence
intention	empathy	irrelevant	reading	risk
read	digits	congruency	memory	reversal
nouns	digit	nonverbal	difficulty	negative
difficulty	memory	pseudoword	semantically	stress
masked	visuomotor	money	scene	craving
orthographic	working	bottomup	categorical	threat
photographs	autism	fluency	past	rule
retrieved	attentional	words	word	integration

card	strategies	stories	motivation	aversive
category	actions	explicit	scenarios	effort
ability	difficulty	serial	mental	reactivity
phonology	risk	nback	names	associative
languages	name	saccade	verbal	cues
familiarity	recall	engagement	languages	losses
skills	text	incongruent	trait	fearful
empathy	rules	working	strategy	negatively
success	feelings	picture	probabilities	oddball
imagine	incorrect	rules	rehearsal	spontaneous
virtual	gonogo	arithmetic	monitoring	reinforcement
outcomes	masked	solving	manipulation	incentive
rules	thought	engaged	conflict	inhibitory
intelligence	nonverbal	prime	irrelevant	thoughts
eye	irrelevant	cards	experiences	self
digit	loss	speakers	adolescents	incorrect
interference	schizophrenia	competition	knowledge	emotions
autism	improvement	verbal	engagement	conditioned
topdown	success	rule	personal	intention
strategies	knowledge	game	outcome	awareness
personal	depression	masked	familiarity	personal
nonwords	decision	associations	masked	nonwords
nback	objects	reward	subtraction	encoding
meaning	future	demand	shortterm	anticipatory
expressions	adolescents	face	animals	1back
abilities	retrieved	eating	ambiguity	selection
self	rest	encoding	engaged	intentional
demand	demand	difficulty	adolescence	disgust
text	schizophrenic	smoking	contexts	memory
working	interference	search	drug	retrieved
recall	negatively	classification	social	recollection
monitoring	adaptation	digit	person	competition
strategy	retention	children	explicit	shifting
familiar	load	grammatical	drugs	planning
effort	calculation	familiar	shapes	gain
conflict	experiences	goal	selection	pseudowords
relational	aging	losses	associations	habituation
actions	judgment	categorical	search	development
digits	unfamiliar	executive	identity	2back
recognition	negative	story	english	shock
retention	strategy	dopaminergic	goal	concentration
irrelevant	identity	reinforcement	unfamiliar	error
phonological	topdown	expressions	masking	sexual
pairs	taskrelated	learning	concentration	phonology
developmental	episode	episode	choice	ambiguity
memory	tool	child	mentalizing	goal
faces	incongruent	covert	selfreported	taskirrelevant
noun	goal	sentence	encoding	sequential
knowledge	contexts	selfreported	orthographic	approach

impulsivity	theory	expectancy	schizophrenia	disorder
engaged	names	sentences	learning	violation
colour	masking	scenarios	violation	category
cued	abilities	comprehension	numbers	relational
distractor	losses	manipulation	developmental	positive
incorrect	preparatory	errors	aging	suppression
goal	control	longterm	classification	experience
sentences	choice	memory	perspective	addiction
adolescents	experience	person	personality	semantic
language	mirror	colors	imagery	abilities
saccades	intelligence	correct	correct	contextual
thought	emotion	perceptual	lexical	happy
categorization	planning	eye	ability	depressed
theory	conscious	decisionmaking	imagine	cueing
explicit	subtraction	tool	intentions	violations
emotion	association	ambiguous	scenes	cue
episode	picture	conflict	phasic	attentional
imagined	reading	childhood	topdown	depression
adaptation	target	moral	objects	musical
emotions	switching	aging	depression	associations
fluency	work	experiences	loss	episode
face	avoidance	videos	contextual	mentalizing
priming	inhibition	vision	gain	impulsivity
encoded	cue	past	future	scenarios
masking	errors	developmental	adaptation	longterm
future	familiar	knowledge	language	conflict
loss	developmental	maintenance	read	distractor
rule	cueing	skills	words	expression
errors	selection	masking	meaning	unpleasant
contextual	eyes	addiction	phonological	ability
words	visual	distractor	error	gonogo
improvement	drugs	outcome	traits	retention
orienting	distractor	animal	sentences	subjective
feelings	expectancy	loss	children	interference
scenes	finger	facial	fact	automatic
rest	relational	error	competition	mood
experiences	practice	movie	child	language
experience	attention	selection	craving	schizophrenia
visuomotor	emotions	interference	abilities	order
linguistic	skills	audiovisual	conditioned	selfreferential
tracking	engaged	gain	naming	rating
verbal	pictures	adaptation	expectancy	errorrelated
mirror	longterm	craving	illness	contexts
tool	positive	saccades	negatively	go
aging	shifts	learned	persons	maintenance
choice	affective	contexts	calculation	sadness
calculation	fact	personal	photographs	words
selection	learning	tracking	association	childhood
visual	spatially	shortterm	disorder	serial

bottomup	autistic	reversal	tracking	avoidance
eyes	rotation	novelty	childhood	decisions
congruent	colour	errorrelated	feelings	paired
visually	rule	congruent	attentional	switching
vision	motion	autistic	errors	music
fact	ratings	crossmodal	grammatical	adaptation
valence	subjective	fact	tool	syntactic
english	color	numbers	intentional	anticipation
rotation	gaze	taskirrelevant	nonwords	sensorimotor
gaze	number	rehearsal	colour	irrelevant
perceptual	depressed	motivation	thought	orientation
negatively	valence	social	number	play
control	depressive	tom	training	taskrelated
child	familiarity	visually	pairs	monitoring
target	disorder	experience	depressive	recognition
negative	image	attentional	cued	grammatical
saccadic	eating	associative	learned	linguistic
correct	orientation	text	name	outcomes
writing	go	choice	negative	regulation
sleep	word	happy	spatially	anger
selfreported	motor	abilities	experience	depressive
attentional	correct	shifts	category	selfreported
work	visually	outcomes	control	manipulation
association	category	conscious	congruency	prime
practice	encoded	color	conscious	preparatory
longterm	scenes	contextual	colors	outcome
grammatical	order	males	violations	wm
planning	locations	neutral	switching	demand
expectancy	taskirrelevant	practice	interference	correct
schizophrenic	win	probabilities	practice	shortterm
load	automatic	development	probe	delay
go	time	illness	adults	search
encoding	encoding	visual	linguistic	learning
attended	photographs	mental	skills	cued
conscious	verbal	presentations	imagined	pseudoword
males	fingers	number	amount	association
shape	mask	risk	development	listening
risk	classification	shape	work	retrieval
learning	explicit	adults	women	shifts
spatially	regulation	load	conditioning	acquisition
videos	contextual	spatially	taste	imagined
taskrelated	colors	women	shifts	dyslexia
comprehension	response	violations	categorization	autobiographical
affective	selfreported	numerical	incongruent	story
taskirrelevant	sequence	written	rotation	discrimination
number	vision	probe	taskrelated	perceived
positive	spontaneous	acquisition	response	engagement
manipulation	images	autobiographical	empathy	calculation
adults	written	preference	schizophrenic	neutral

written	velocity	gaze	eye	subsequent
order	smoking	attended	theory	autism
depressed	congruent	discrimination	picture	aphasia
attention	males	gonogo	order	stories
facial	manipulation	negatively	recognition	practice
image	tapping	thinking	prime	ratings
motion	error	anger	distractor	phonological
colors	anxiety	intentional	syntactic	nonverbal
speakers	preparation	perspective	images	aging
classification	illness	switching	virtual	trait
pseudowords	physical	association	time	gaze
finger	subsequent	adolescence	age	future
mask	development	subtraction	mask	space
cueing	play	response	cueing	working
time	associations	sex	target	lexical
automatic	read	mask	mood	somatotopic
tapping	action	target	subjective	expectancy
response	reward	adolescents	saccade	errors
color	conditioned	personality	acquisition	reward
anger	amount	mirror	familiar	preparation
subtraction	nogo	monitoring	speakers	load
gonogo	shape	expectation	image	encoded
images	categorical	violation	play	numerical
associations	training	images	attribution	trained
ratings	cues	subsequent	saccades	painful
training	face	order	fluency	decision
orientation	age	theory	cues	orthographic
error	reaching	drug	ratings	visuospatial
development	simulation	training	counting	sensory
illness	hand	speech	food	masked
sequence	numbers	drugs	associative	sequence
categorical	spatial	amount	expectation	traits
numbers	adults	ability	reversal	thought
age	pairs	expression	inhibition	win
subsequent	semantic	time	subsequent	solving
depressive	verb	production	visual	pain
ambiguous	rating	cues	face	sensation
affect	drug	age	attention	velocity
spontaneous	acquisition	card	effort	strategies
sadness	voluntary	sadness	color	voluntary
implicit	child	attention	mirror	attend
conditioned	numerical	spatial	phonology	attention
motor	heart	awareness	intention	nogo
schizophrenia	affect	motion	uncertainty	repetitions
covert	space	thought	affective	somatosensory
presentations	words	implicit	self	priming
disorder	dopaminergic	image	avoidance	orienting
switching	feedback	go	planning	stimulation
acquisition	limb	effort	orienting	expectation

females	inhibitory	depressed	pictures	crossmodal
play	serial	uncertainty	animal	rehearsal
locations	outcome	orientation	positive	syllable
depression	probe	rotation	sequence	classification
cue	money	taste	written	visuomotor
movie	perceptual	future	depressed	verbal
inhibition	repetition	repetition	congruent	mask
serial	approach	females	autism	overt
emotionally	hands	male	approach	execution
repetition	expressions	dyslexia	males	nociceptive
amount	females	mood	stress	phasic
male	nouns	control	actions	oral
shifts	faces	family	salience	scenes
shortterm	male	sexual	endogenous	reaching
cues	readers	virtual	cue	monetary
empathic	videos	threat	spatial	articulatory
hand	sensorimotor	imagery	motion	rest
space	bottomup	cueing	anticipation	distractors
integration	motivation	disorder	covert	reasoning
physical	foot	concentration	preference	attribution
probe	attend	conditioning	emotion	simulation
action	empathic	paired	visually	skills
neutral	women	trait	ambiguous	adolescence
spatial	meaning	space	anxiety	pressure
children	sexual	play	bottomup	noun
family	recognition	work	space	naming
violation	fluency	positive	sexual	verb
subjective	awareness	autism	repetition	subtraction
regulation	mood	mentalizing	valence	sequences
simulation	attended	sequence	gonogo	name
approach	presentations	automatic	delay	family
rating	naming	imagine	neutral	distress
attend	execution	negative	rating	colour
spoken	probabilities	schizophrenic	improvement	demands
stress	stress	persons	affect	voice
foot	movements	approach	motor	audiovisual
threat	shortterm	phasic	physical	response
disgust	movie	depressive	writing	nouns
sentence	endogenous	angry	faces	noxious
production	categorization	affect	females	grasping
rehearsal	sadness	ratings	visuomotor	rewards
ambiguity	implicit	empathic	male	prosody
gesture	emotionally	orienting	vision	digit
avoidance	painrelated	endogenous	automatic	masking
childhood	covert	empathy	sentence	arithmetic
discrimination	salience	position	hand	expressions
hands	distress	repetitions	priming	olfactory
grasping	learned	integration	family	gesture
probabilities	aversive	food	orientation	english

reaching	monetary	saccadic	autistic	spoken
dyslexia	neutral	subjective	finger	work
nogo	counting	perception	awareness	multisensory
women	sex	locations	emotions	repetition
awareness	childhood	salience	heart	dopaminergic
distress	family	inhibition	perceptual	saccade
syntactic	repetitions	physical	action	position
anxiety	expectation	cued	paired	feedback
eating	position	heart	locations	sensations
velocity	rehearsal	thoughts	go	languages
movements	noun	hand	body	saccades
heart	children	emotion	sequences	rules
fingers	gain	emotions	presentations	topdown
drugs	integration	counting	sequential	digits
position	body	disgust	production	phonetic
sexual	concentration	feelings	rest	adolescents
salience	priming	schizophrenia	sex	stop
limb	violation	gesture	shape	vocal
unpleasant	taste	taskrelated	novelty	vision
noise	delay	delay	implicit	color
preference	facial	intentions	comprehension	attended
perception	language	causality	videos	sex
repetitions	sensory	self	position	painrelated
syllables	semantically	rating	movie	acoustic
counting	suppression	anxiety	pleasant	fluency
animals	conditioning	valence	temperature	game
losses	discrimination	affective	saccadic	taste
mood	stop	intention	dyslexia	bottomup
preparatory	threat	calculation	expressions	training
sex	preference	syllables	integration	explicit
aversive	uncertainty	body	feedback	scene
expression	rewards	improvement	sadness	auditory
preparation	disgust	depression	simulation	reading
conditioning	anticipation	cue	empathic	writing
body	unpleasant	stress	fingers	adults
feedback	sequences	action	pseudowords	actions
voluntary	nonwords	sequential	unpleasant	production
learned	animals	conditioned	gesture	written
expectation	anger	suppression	inhibitory	amount
drug	ambiguous	trained	expression	presentations
inhibitory	noise	olfactory	olfactory	visual
outcome	trained	traits	anger	shapes
fearful	grasping	noise	disgust	rotation
numerical	perception	motor	spoken	illness
aphasia	lexical	avoidance	aversive	sleep
paired	languages	sequences	repetitions	male
associative	associative	fearful	trained	read
sequences	pain	anticipation	shock	tapping
syllable	shock	auditory	discrimination	image

sensory	gesture	shock	regulation	target
reward	english	pleasant	threat	noise
concentration	paired	aphasia	emotionally	fact
execution	stimulation	finger	gaze	sentence
painrelated	expression	visuomotor	facial	sounds
suppression	heat	planning	taskirrelevant	tracking
sensorimotor	perceived	emotionally	noise	word
trained	sequential	distress	eyes	names
pleasant	writing	inhibitory	reaching	readers
money	animal	movements	preparatory	money
delay	addiction	actions	nogo	females
speech	violations	attend	anticipatory	colors
pseudoword	painful	unpleasant	happy	sentences
animal	orthographic	reaching	distress	facial
smoking	anticipatory	oddball	sleep	movements
gain	sentences	articulatory	tapping	images
taste	pleasant	eyes	heat	movie
auditory	production	reactivity	attended	heat
motivation	reinforcement	overt	preparation	heart
violations	sensations	phonetic	suppression	saccadic
happy	skin	attribution	painrelated	gestures
audiovisual	food	syllable	pseudoword	action
perceived	phonological	imagined	attend	visually
stop	ambiguity	preparatory	movements	counting
crossmodal	linguistic	rest	perceived	nonpainful
endogenous	nonpainful	feedback	foot	prosodic
monetary	unpleasantness	stop	stop	shape
stimulation	somatosensory	perceived	syllables	tactile
shock	phasic	voice	perception	probe
uncertainty	speakers	sounds	sensory	locations
angry	dyslexia	aversive	execution	hearing
sequential	syllable	gestures	unpleasantness	number
win	oral	simulation	hands	speech
dopaminergic	grammatical	sound	skin	motion
pain	novelty	anticipatory	auditory	speakers
gestures	auditory	sleep	noxious	persons
overt	noxious	selfreferential	syllable	numbers
reactivity	sensation	regulation	spontaneous	physical
oral	comprehension	fingers	habituation	pitch
food	incentive	mouth	aphasia	temperature
unpleasantness	habituation	preparation	overt	pairs
painful	fearful	sensory	oral	pictures
novelty	happy	listening	sensorimotor	age
anticipation	reactivity	unpleasantness	crossmodal	syllables
tactile	tactile	habituation	limb	tones
habituation	overt	oral	angry	sound
heat	pseudowords	grasping	painful	picture
addiction	muscle	nogo	stimulation	tool
skin	syllables	temperature	reactivity	drug

rewards	olfactory	execution	oddball	text
reinforcement	crossmodal	foot	pain	person
sensations	temperature	multisensory	grasping	letters
anticipatory	music	stimulation	speech	motor
music	nociceptive	tapping	velocity	males
somatosensory	oddball	painrelated	fearful	animals
sensation	pressure	hearing	music	limb
muscle	craving	spontaneous	voluntary	eating
phasic	sentence	skin	nociceptive	eyes
noxious	speech	pressure	pressure	eye
nonpainful	spoken	noxious	gestures	videos
mouth	angry	lip	nonpainful	drugs
oddball	gestures	pain	sensation	faces
temperature	aphasia	painful	tactile	letter
olfactory	audiovisual	velocity	sounds	children
listening	phonology	heat	musical	photographs
articulatory	pseudoword	hands	audiovisual	mirror
voice	mouth	prosody	articulatory	smoking
pressure	muscles	tactile	listening	objects
musical	syntactic	music	mouth	women
multisensory	musical	ear	sound	face
incentive	multisensory	musical	sensations	card
sound	lip	tones	ear	body
lip	somatotopic	limb	somatosensory	child
phonetic	tones	voluntary	muscle	animal
nociceptive	pitch	nociceptive	voice	muscle
craving	listening	sensorimotor	hearing	food
sounds	sound	vocal	somatotopic	muscles
muscles	hearing	nonpainful	tones	finger
hearing	articulatory	somatotopic	phonetic	ear
somatotopic	sounds	prosodic	multisensory	fingers
tones	voice	acoustic	pitch	skin
pitch	ear	sensation	prosody	lip
vocal	vocal	sensations	vocal	foot
ear	phonetic	pitch	lip	hand
prosody	prosody	somatosensory	acoustic	mouth
prosodic	acoustic	muscle	muscles	cards
acoustic	prosodic	muscles	prosodic	hands
